# Perceived healthiness of sugary drinks and related social norms among adults in five countries: evidence from the International Food Policy Study

**DOI:** 10.1186/s12937-024-01063-8

**Published:** 2025-01-29

**Authors:** Virginie Drolet-Labelle, Christine M. White, Jean Adams, Sharon I. Kirkpatrick, Alejandra Jáuregui, Lilia S. Pedraza, Véronique Provencher, Gary Sacks, James F. Thrasher, Gabriela C. Armendariz, Simón Barquera, David Hammond, Lana Vanderlee

**Affiliations:** 1 École de nutrition, Faculté des sciences de l’agriculture et de l’alimentation (FSAA), Université Laval, 2440, boulevard Hochelaga, Québec, Québec G1V 0A6 Canada; 2 Centre Nutrition, santé et société (NUTRISS), Université Laval, Québec, Québec G1V 0A6 Canada; 3 School of Public Health Sciences, University of Waterloo, Waterloo, ON N2L 3G1 Canada; 4 MRC Epidemiology Unit, University of Cambridge, Cambridge, CB2 1TN UK; 5 Center for Health and Nutrition Research, Instituto Nacional de Salud Pública, Morelos, Cuernavaca, 62100 Mexico; 6 Global Obesity Center, Deakin University, Victoria, Burwood, VIC 3125 Australia; 7 Department of Health Promotion, Education & Behavior, Arnold School of Public Health, University of South Carolina, Columbia, SC 29208 USA

**Keywords:** Sugary drinks, Sugar-sweetened beverages, Perceived healthiness, Social norms, Food policy

## Abstract

**Background:**

A better understanding of correlates of sugary drink consumption is essential to inform public health interventions. This study examined differences in perceived healthiness of sugary drinks and related social norms between countries, over time, and sociodemographic groups and associations with sugary drink intake.

**Methods:**

This study used annual cross-sectional data from the International Food Policy Study from 2018 to 2021 in Australia, Canada, the United Kingdom, the United States, and Mexico. Analyses examined perceived healthiness of eight beverage types and two types of perceived social norms (descriptive, injunctive) that discourage sugary drink consumption. The 24-item Beverage Frequency Questionnaire was used to estimate beverage intake in the past 7 days. Logistic regression models examined trends over time in odds of perceiving each beverage type as unhealthy and agreeing with social norms discouraging sugary drink consumption, across countries and sociodemographic characteristics. Negative binomial regressions examined associations between perceived healthiness, social norms and consumption.

**Results:**

Energy drinks, regular soft drinks, and diet soft drinks were most frequently perceived as unhealthy in all countries, while water and 100% juice were least frequently perceived as unhealthy. Participants in Mexico had higher odds of perceiving 100% juice, chocolate milk, and iced tea as unhealthy in 2021 compared to 2018 (AOR = 1.71 99%CI 1.10–2.64; AOR = 2.69, 99%CI 1.70–4.26; AOR = 1.79, 99%CI 1.15–2.76; respectively), with little change in other countries. Agreement with social norms discouraging consumption of sugary drinks was higher in Mexico than in other countries. Trends in social norms over time were mostly stable, except in Mexico where participants had higher odds of agreeing with both norms in 2020 compared to 2018 (AOR = 1.27, 99%CI 1.09–1.48 for a descriptive norm and AOR = 1.27 99%CI 1.09–1.49 for an injunctive norm). In most countries, perceiving a beverage as unhealthy and agreeing with social norms discouraging consumption of sugary drink were associated with lower sugary drink consumption, with varying strength of associations across countries and beverage types.

**Conclusions:**

Shifts over time in social norms and perceived healthiness observed in Mexico and associations with intake of sugary drinks in most countries suggest that targeted interventions to change norms and perceptions could help reduce sugary drink consumption.

**Supplementary Information:**

The online version contains supplementary material available at 10.1186/s12937-024-01063-8.

## Introduction

Consumption of sugary drinks is a significant public health concern as it contributes to excess free and added sugar intake [1–4]. High intakes of sugary drinks and free sugars are associated with an increased risk of noncommunicable diseases, such as cardiovascular disease [5], type 2 diabetes [6], cancer [7], and dental caries [8], with concomitant economic implications for the health care system [9]. Definitions of sugary drinks and sugar-sweetened beverages (SSBs) vary in the literature, but sugary drinks are usually defined as beverages containing free sugars, i.e., SSBs and 100% fruit juices [9, 10].

In recent years, several public health initiatives, including marketing restrictions [11], taxes [12], and mass media campaigns [13, 14], have targeted SSBs, in an effort to reduce consumption. However, while consumption of some beverage categories, such as traditional soft drinks and 100% juice, may be declining [15–18], the popularity of novel drink categories, including iced tea, flavored sweetened water, and energy drinks, continues to rise [15, 17–20].

Research has identified several factors associated with SSB consumption, including sociodemographic characteristics such as younger age, male sex and gender, lower income and education, living with overweight and obesity [16, 21–24], and other dietary and environmental factors [22–24], while demographic and behavioral correlates of consumption for 100% juice are less consistent [16, 25, 26]. Other potentially important individual-level factors that are shaped by sociocultural and policy environments, including beliefs about sugary drinks, have been less frequently studied. For example, social norms are an important determinant of behavior [27, 28] and are central to some behavior change theories [29, 30]. Most studies on social norms have focused on descriptive norms (what others do) related to SSBs [31–34], and, to a lesser extent, on injunctive norms (perceived approval/disapproval of others) [34]. Overall, evidence suggests that perceiving that others drink SSBs is associated with higher individual intake [31–34]. Perceived healthiness of a food has been shown to influence dietary behaviors [35] and the limited studies available have demonstrated wide variations in perceptions of healthiness by beverage type [36–38]. However, differences in perceptions by sociodemographic characteristics, for a range of beverages, including novel drink categories more seldomly consumed, have not been explored. In addition, few studies have examined whether social norms and perceived healthiness, related specifically to sugary drinks, vary across countries with different food policy environments. Understanding the correlates of sugary drink consumption and how they vary across sociodemographic groups and countries could help develop targeted interventions to promote healthier beverage choices while addressing social inequalities.

This study aimed to determine: 1) how perceived healthiness and social norms toward sugary drinks vary over time, across sociodemographic groups and countries; and 2) how perceived healthiness and social norms are associated with sugary drink intake.

## Methods

### Study design

Cross-sectional survey data were analyzed from the 2018-2021 waves of the International Food Policy (IFPS), which includes 5 countries with varying food environments, policies and cultures and thus provide unique opportunities for comparison [39]. Adults aged ≥ 18 years residing in Australia, Canada, Mexico, the United Kingdom (UK), and the United States (US) were recruited through the Nielsen Consumer Insights Global Panel and their partners’ panels using non-probability sampling methods. Invitations with unique survey access links were sent to a random sample of available panelists in each country. Quotas for age, sex, and language (when relevant) were applied to facilitate the recruitment of samples that were more representative of each country’s population, based on national census data in each country. Self-administered surveys were conducted online using Alchemer Online Survey Software. All participants provided consent prior to completing the survey and received remuneration following their panel’s usual incentive structure [40]. The study received ethical approval by a University of Waterloo Research Ethics Committee (REB #30829) and Université Laval Ethics Committee (#2021-318 Phase II A-2 R-2 / 11-12-2023).

### Survey measures

#### Perceived healthiness of sugary drinks

Perceived healthiness of sugary drinks was assessed with the question: “In your opinion, how unhealthy or healthy is this type of drink?” with response options on a 7-point Likert scale (“Very unhealthy”, “Unhealthy”, “A little unhealthy”, “Neither healthy nor unhealthy”, “A little healthy”, “Healthy”, “Very healthy”). Participants were asked to respond for regular soft drinks (Coca-Cola™) and another randomly assigned branded beverage from the following 8 categories: diet soft drinks, 100% juice, energy drinks, water, specialty coffee, sports drinks, chocolate milk, or iced tea. Randomization to the second beverage category reviewed was conducted within each country sample. White milk was added in 2019 but was not analyzed in the current study. The product images shown to participants for the perceived healthiness questions can be found in Figure S1.These images matched brands and products commonly available in each country and remained the same across all waves, except in Mexico, where front-of-pack (FOP) warning labels were added in 2020, in line with national regulations [41]. Relative perceived healthiness was measured as the difference between the initial 7-point Likert scale healthiness rating of the regular soft drink and the rating of the additionally assigned drink.

#### Perceived social norms for sugary drinks

Participants were asked to respond to two statements, including “People important to me TRY NOT to drink SUGARY DRINKS” (a descriptive norm) and “People important to me THINK I SHOULD NOT drink SUGARY DRINKS” (an injunctive norm) (underlining and capital letters were used in the wording for emphasis), using a 5-point Likert scale, (“Strongly agree”, “Agree”, “Neither agree nor disagree”, “Disagree”, "Strongly disagree”) in addition to “Don’t know” and “Refuse to answer”. The underlined and capitalized words were changed for the 2021 survey, which may have influenced responses; therefore, social norm data from 2021 were excluded from analyses. Before answering the questions about social norms, participants were given the following definition: “Sugary drinks are drinks that contain added sugar, like pop, fruit drinks, sports drinks, energy drinks, chocolate milk, and specialty coffees that have added sugar”. Notably, this did not include 100% fruit juice, which is not consistent with most definitions of sugary drinks.

#### Sugary drink intake

Sugary drink consumption was estimated using the Beverage Frequency Questionnaire, which assesses consumption of 20 non-alcoholic and 4 alcoholic beverages and has demonstrated reasonable validity among Canadian young adults [42]. Participants were asked to report the number of drinks consumed with the following question: “During the past 7 days, how many drinks did you have in each category below?”. A sugary drink intake variable was created by summing the number of beverages consumed from each category that contained added sugars (regular soft drinks, sports drinks, energy drinks, chocolate/flavored milk, fruit drinks, flavored/vitamin waters, and sweetened smoothies/protein shakes/drinkable yogurts) but did not include 100% fruit juice, which was consistent with the definition of sugary drinks that was provided to participants. Specialty coffee and coffee/tea with sugar were not included in the grouped variable as these measures were changed over time and thus cannot be used for longitudinal comparisons. Alcoholic beverages were also not included in this analysis.

Participants who reported 70 to 100 beverages per week for a single drink category were recoded as 70 drinks/week (for each of those applicable drink categories). Participants were excluded from the BFQ analyses due to data quality concerns if they reported more than 100 beverages per week for a single drink category, there were inconsistencies in reporting numbers of drinks (e.g., reporting no drinks consumed all week, conflicting responses indicating no drinks consumed while also reporting specific drinks consumed, repetitive/sequential entries across all categories, non-numerical/nonsensical entries), missing information on the size of beverage consumed, the total volume consumed was reported as greater than 49 liters per week, and/or the total volume consumed, excluding water was reported as greater than 36 liters per week [40].

#### Sociodemographic characteristics

Sociodemographic variables selected *a priori* as covariates of interest included age, ethnicity, sex at birth, country, income, education, and the presence of a child under 18 years at home. Ethnicity was assessed using country-specific race/ethnicity categories and was analyzed as a binary variable (majority ethnicity/minority ethnicity) to facilitate comparisons across countries. Perceived income adequacy was assessed with the question, “Thinking about your total monthly income, how difficult or easy is it for you to make ends meet?”, with response options on a 5-point scale, from “Very difficult” to “Very easy”, which allows for better comparisons across countries than absolute household income [43]. Data were recoded as “Low income adequacy” (Very difficult/Difficult/Neither easy nor difficult to make ends meet) and “High income adequacy” (Easy/Very Easy to make ends meet). Education was assessed with the question, “What is the highest level of formal education that you have completed?”. Responses were categorized into three levels, including low (high school diploma or less), medium (technical diploma or some post-secondary qualifications), and high education (university degree or higher), to facilitate comparisons across countries.

For all variables (perceived healthiness, social norms, sociodemographic characteristics, and frequency of beverage consumption), participants who answered, “Refuse to answer” or “Don’t know” were considered missing and were excluded from all analyses. Participants who experienced a technical glitch in the survey programming (*n*=289), whereby they were not randomized and were asked the perceived healthiness question for all beverages, were also excluded, as were those who were randomly assigned to a glass of white milk in 2021, as this was not analyzed in the current study.

### Statistical analysis

All analyses were performed with SAS Studio V.3.8. Data were weighted with post-stratification sample weights constructed using a raking algorithm with population estimates from census data in each country based on known population age, sex, region, ethnicity (except in Canada), and education level (except in Mexico) [40].Weights were rescaled to the final sample size. Descriptive estimates for sociodemographic, perceived healthiness, and social norms are reported as unadjusted weighted percentages or counts, by country and year which were calculated using SAS survey procedures.

In order to model factors that may be more likely to be associated with reduced consumption of sugary drinks (which is important from a public health perspective), continuous Likert scale responses for perceived healthiness were recoded into a binary variable of “Unhealthy” (very unhealthy/unhealthy/a little unhealthy) and “Not unhealthy” (neither healthy nor unhealthy/a little healthy/healthy/very healthy), and social normal responses were recoded into binary variables of “Agree” (strongly agree/agree) and “Not Agree” (neither agree nor disagree/disagree/strongly disagree). Sensitivity analyses were conducted to compare the use of a binary variable compared to a continuous scale and the pattern of results did not change (data not shown).

Logistic regressions weighted and adjusted for sociodemographic variables, including age, ethnicity, sex, perceived income adequacy, education, the presence of a child under 18 at home, and year were used to assess differences between countries in the odds of perceiving each beverage category as unhealthy (among the subsamples randomly assigned to each beverage), as well as the odds of agreeing with the descriptive and the injunctive norms. Similar regressions, stratified by country, examined differences by year and sociodemographic variables.

Adjusted negative binomial regressions, weighted and stratified by country and beverage, were used to examine the association between each correlate and sugary drink intake. For perceived healthiness, perception of each sugary beverage type was linked with intake of each drink. For example, models examined the association between perceived healthiness of regular soft drinks and the number of times a soft drink was consumed in the past week. Separate models were also used to examine the associations between each of the descriptive and injunctive norms and the number of times any sugary drink was consumed in the past seven days. Adjusted odds ratios (AORs) and incident risk ratios (IRRs) are reported with 99% confidence intervals to account for the large sample size and multiple comparisons examined. AORs and IRRs are interpreted using effect size (i.e., the magnitude of the association), as follows: <1.5 = very small, 1.5 to 2 = small, 2 to 3 = medium, > 3 = large [44].

## Results

### Sample characteristics

Of the 86 683 adults who completed the IFPS surveys, 14 182 (16.4%) participants had missing data for sociodemographic variables (*n*=1036 for perceived income adequacy, *n*=1045 for ethnicity, *n*=379 for education, *n*=179 for the presence of children at home), the two correlates of interest (*n*=2545 for perceived healthiness measures; *n*=6016 for social norms), or the beverage intake variables (*n*=8978; numbers may sum to greater than 14 182 due to missing data for multiple variables), and 2134 (2.5%) were randomized to conditions not examined in the current study. The final analytic sample included 70 367 participants (Australia=13 657; Canada=14 204; Mexico=14 220; UK=14 001; US=14 285). Weighted sample characteristics are shown in Table 1.

**Table 1:** Weighted sociodemographic characteristics across countries (2018–2021)^a^

	All countries (*n* = 70 367)	Australia (*n* = 13 657)	Canada (*n* = 14 204)	Mexico (*n* = 14 220)	UK (*n* = 14 001)	US (*n* = 14 285)
	n (%)					
**Survey year**						
2018	18 683 (27)	3403 (25)	3642 (26)	3531 (25)	4286 (31)	3821 (27)
2019	17 708 (25)	3551 (26)	3491 (25)	3800 (27)	3281 (23)	3585 (25)
2020	18 047 (26)	3564 (26)	3581 (25)	3701 (26)	3405 (24)	3796 (27)
2021	15 929 (23)	3139 (23)	3490 (25)	3188 (22)	3029 (22)	3083 (22)
**Sex**						
Male	34 085 (48)	6680 (49)	7018 (49)	6784 (48)	6776 (48)	6828 (48)
Female	36 282 (52)	6977 (51)	7186 (51)	7436 (52)	7225 (52)	7457 (52)
**Age group**						
18–29 years	15 374 (22)	2825 (21)	2762 (19)	4148 (29)	2698 (19)	2941 (21)
30–44 years	18 723 (27)	3644 (27)	3566 (25)	4494 (32)	3489 (25)	3529 (25)
45–59 years	18 187 (26)	3331 (24)	3584 (25)	4064 (29)	3584 (26)	3624 (25)
≥60 years	18 083 (26)	3856 (28)	4291 (30)	1514 (11)	4230 (30)	4191 (29)
**Ethnicity**^**b**^						
Majority	54 462 (77)	10 164 (74)	11 213 (79)	11 375 (80)	12 459 (89)	9252 (65)
Minority	15 905 (23)	3493 (26)	2991 (21)	2845 (20)	1542 (11)	5033 (35)
**Education**^**c**^						
Low	29 396 (42)	5645 (41)	5836 (41)	2976 (21)	6912 (49)	8027 (56)
Medium	15 549 (22)	4399 (32)	4791 (34)	1891 (13)	3028 (22)	1441 (10)
High	25 422 (36)	3614 (26)	3577 (25)	9352 (66)	4061 (29)	4817 (34)
**Income adequacy**^**d**^						
Low	47 069 (67)	8572 (63)	9216 (65)	11 843 (83)	8335 (60)	9103 (64)
High	23 298 (33)	5085 (37)	4988 (35)	2377 (17)	5666 (40)	5182 (36)
**Children (< 18y) at home**						
No	48 491 (69)	10 025 (73)	11 109 (78)	7049 (50)	10 174 (73)	10 134 (71)
Yes	21 876 (31)	3632 (27)	3095 (22)	7171 (50)	3827 (27)	4151 (29)

^a^ Percentages (%) and frequencies (n) are weighted

^b^ Ethnicity was assessed using country-specific race/ethnicity categories and was analyzed as a binary variable (majority/minority). Ethnicity categories were recoded as follows: (1) Australia majority = only speaks English at home, minority = speaks a language other than English at home or indicated they are aboriginal or Torres Straight Islander; (2) Canada majority = White, minority = other ethnicity; (3) Mexico majority = Non-indigenous, minority = indigenous; (4) UK majority = White, minority = other ethnicity; (5) US majority = White, minority = other ethnicity

^c^ Participants were asked: “What is the highest level of formal education that you have completed?”. Responses were then classified into 3 levels, i.e. low (high-school diploma or less), medium (technical diploma or some post-secondary qualifications), and high education (university degree or higher)

^d^ Participants were asked: “Thinking about your total monthly income, how difficult or easy is it for you to make ends meet?”, “Very difficult”, “Difficult” and “Neither easy nor difficult” were combined as low income adequacy, and “Easy” and “Very easy” as high income adequacy

### Perceived healthiness of beverages

**Fig. 1:**
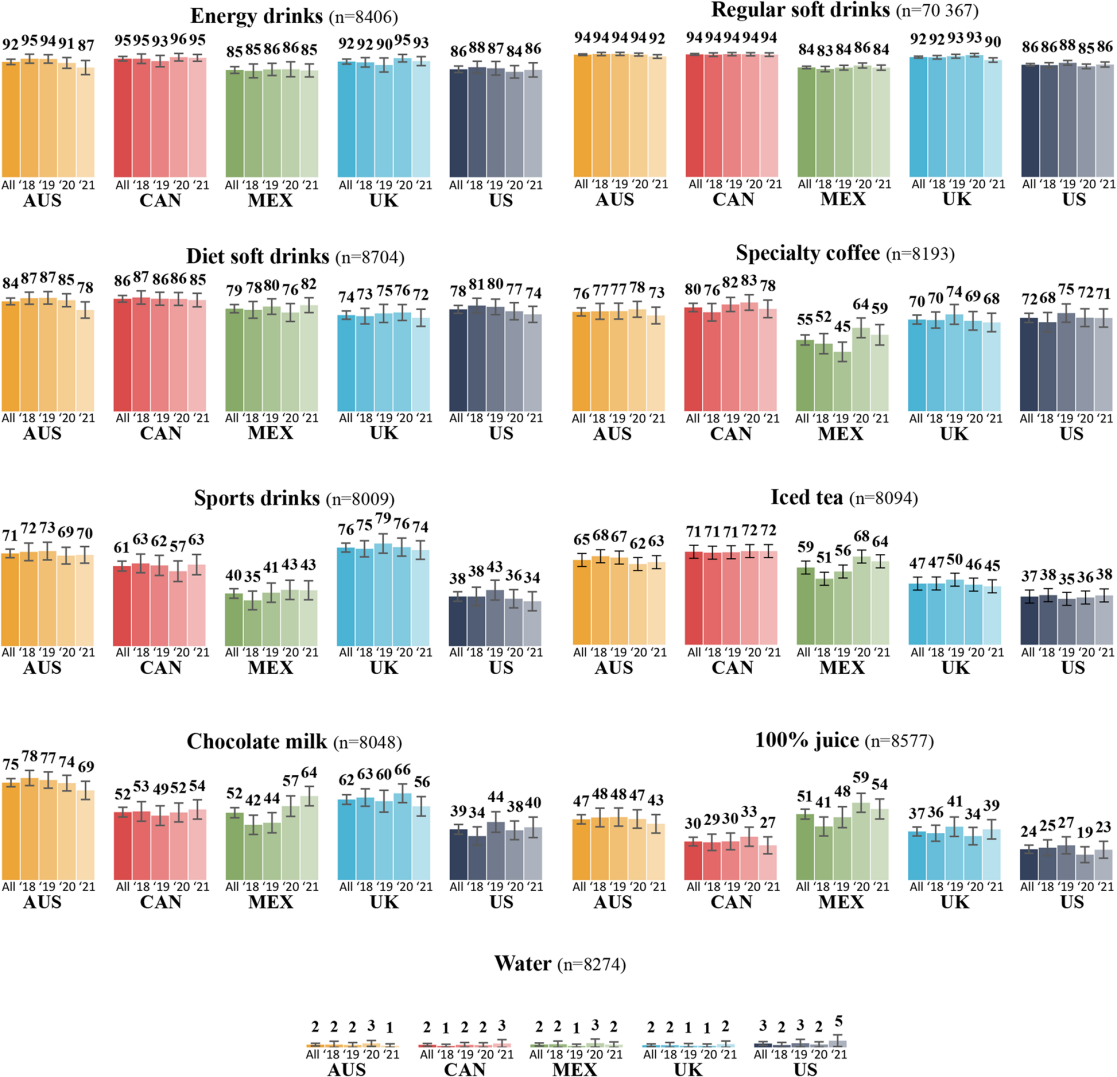
Weighted percentages of participants reporting each beverage as unhealthy across all years combined and for each year^a^. Legend: ^a^Participants were asked: “In your opinion, how unhealthy or healthy is this type of drink?” using a 7-point Likert-type scale, with response options “A little unhealthy”, “Unhealthy” and “Very unhealthy” categorized as “Unhealthy”. Error bars represent 99% confidence intervals. Abbreviations: AUS = Australia, CAN = Canada, MEX = Mexico, UK = United-Kingdom, US = United-States, All = All countries, ‘18 = 2018, ‘19 = 2019, ‘20 = 2020, ‘21 = 2021

**Fig. 2:**
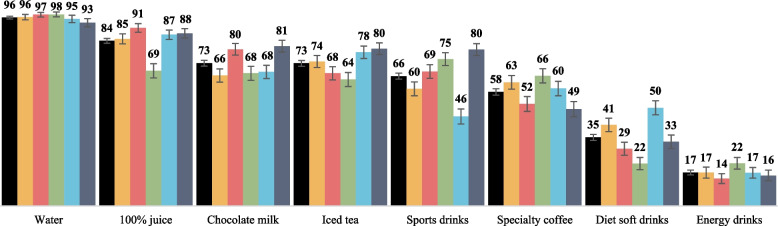
Weighted percentages of participants who rated each beverage as healthier^a^ than regular soft drinks (2018-2021)^b^. Legend:^a^Participants were asked: “In your opinion, how unhealthy or healthy is this type of drink?” using a 7-point Likert-type scale for regular soft drinks and another randomly assigned beverage. ^b^Relative perceived healthiness is the difference between perceived healthiness of regular soft drinks and of the additional beverage category to which the participant was randomized. Error bars represent 99% confidence intervals.  All countries  Australia  Canada  Mexico  UK  US

Figure 1 shows the unadjusted percentages of participants who reported each beverage as unhealthy, by country and year. Overall, the perception that a beverage was unhealthy was most common for energy drinks (range from 85% in the Mexico to 95% in Canada), regular soft drinks (range from 84% in Mexico to 94% in Australia and Canada), and diet soft drinks (range from 74% in the UK to 86% in Canada), and least common for water (about 2% in all countries) and 100% juice (range from 24% in the US to 51% in Mexico). Figure 2 shows the percentage of participants who rated each beverage category as “healthier” compared to the rating of regular soft drinks (e.g., a higher rating on the 7-point Likert scale). Most participants in all countries rated water, 100% juice, chocolate milk, iced tea, sports drinks, and specialty coffee as healthier than regular soft drinks. Figures detailing perceptions of each beverage type on the 7-point Likert scale are available in supplemental files (Figure S2).

In adjusted models, there were mostly very small to moderate differences between countries in perceived healthiness of regular soft drinks, 100% juice, diet soft drinks, and energy drinks, with several large differences noted for sports drinks, chocolate milk, and iced tea (Table 2). Participants in Australia had higher odds of perceiving regular soft drinks, 100% juice, diet soft drinks, and iced tea, as unhealthy compared with those in the UK, the US, and Mexico (except 100% juice in Mexico), and higher odds of perceiving chocolate milk as unhealthy compared to all countries. Participants in the US had lower odds of perceiving all beverages, except specialty coffee, as unhealthy compared to those in Australia, Canada, and the UK (except diet soft drinks in the UK). Participants in Mexico had lower odds of perceiving regular soft drinks, energy drinks, sports drinks, and specialty coffee as unhealthy compared to those in Australia, Canada, and the UK, but greater odds of perceiving 100% juice as unhealthy compared to all countries, except Australia.

**Table 2:** Differences across countries in the perception of each beverage category as “unhealthy” (2018–2021)

	Regular soft drinks^a^(*n* = 70 367)		100% juice (*n* = 8577)		Diet soft drinks (*n* = 8704)		Energy drinks (*n* = 8406)		Sports drinks (*n* = 8009)		Chocolate milk (*n* = 8048)		Iced tea (*n* = 8094)		Specialty coffee (*n* = 8193)	
	AOR(99%CI)	*p*	AOR(99%CI)	*p*	AOR(99%CI)	*p*	AOR(99%CI)	*p*	AOR(99%CI)	*p*	AOR(99%CI)	*p*	AOR(99%CI)	*p*	AOR(99%CI)	*p*
Australia vs. Mexico^b^	2.69 (2.35;3.09)	< 0.001	0.91 (0.73;1.15)	0.297	1.49 (1.11;1.99)	0.001	1.81 (1.22;2.67)	< 0.001	4.03 (3.16;5.12)	< 0.001	3.20 (2.49;4.11)	< 0.001	1.50 (1.19;1.89)	< 0.001	2.92 (2.27;3.75)	< 0.001
Australia vs. UK	1.37 (1.19;1.58)	< 0.001	1.46 (1.19;1.79)	< 0.001	1.93 (1.49;2.50)	< 0.001	1.11 (0.74;1.65)	0.520	0.80 (0.62;1.02)	0.019	1.87 (1.49;2.36)	< 0.001	2.08 (1.67;2.59)	< 0.001	1.41 (1.11;1.80)	< 0.001
Australia vs. US	2.34 (2.06;2.67)	< 0.001	2.90 (2.31;3.65)	< 0.001	1.45 (1.11;1.90)	< 0.001	1.73 (1.20;2.50)	< 0.001	3.93 (3.12;4.94)	< 0.001	4.51 (3.57;5.71)	< 0.001	3.11 (2.48;3.90)	< 0.001	1.15 (0.90;1.48)	0.137
Australia vs. Canada	1.01 (0.86;1.17)	0.926	2.12 (1.71;2.63)	< 0.001	0.86 (0.64;1.15)	0.177	0.70 (0.45;1.11)	0.046	1.60 (1.27;2.01)	< 0.001	2.84 (2.26;3.56)	< 0.001	0.75 (0.60;0.94)	0.001	0.85 (0.66;1.11)	0.117
Mexico vs. UK	0.51 (0.45;0.58)	< 0.001	1.60 (1.28;2.01)	< 0.001	1.30 (0.99;1.70)	0.012	0.61 (0.42;0.89)	0.001	0.20 (0.15;0.26)	< 0.001	0.59 (0.46;0.74)	< 0.001	1.39 (1.10;1.75)	< 0.001	0.48 (0.38;0.62)	< 0.001
Mexico vs. US	0.87 (0.78;0.98)	0.002	3.18 (2.49;4.07)	< 0.001	0.98 (0.74;1.29)	0.817	0.96 (0.68;1.34)	0.743	0.98 (0.77;1.23)	0.787	1.41 (1.12;1.78)	< 0.001	2.08 (1.65;2.62)	< 0.001	0.40 (0.31;0.51)	< 0.001
Mexico vs. Canada	0.37 (0.32;0.43)	< 0.001	2.32 (1.83;2.95)	< 0.001	0.58 (0.43;0.78)	< 0.001	0.39 (0.25;0.60)	< 0.001	0.40 (0.31;0.50)	< 0.001	0.89 (0.70;1.12)	0.191	0.50 (0.39;0.64)	< 0.001	0.29 (0.22;0.38)	< 0.001
UK vs. US	1.71 (1.59;1.94)	< 0.001	1.99 (1.58;2.51)	< 0.001	0.75 (0.59;0.96)	0.003	1.57 (1.09;2.25)	0.001	4.93 (3.84;6.32)	< 0.001	2.41 (1.92;3.02)	< 0.001	1.50 (1.19;1.88)	< 0.001	0.82 (0.64;1.05)	0.036
UK vs. Canada	0.73 (0.63;0.85)	< 0.001	1.45 (1.16;1.81)	< 0.001	0.44 (0.34;0.58)	< 0.001	0.64 (0.40;1.01)	0.013	2.01 (1.56;2.58)	< 0.001	1.51 (1.21;1.89)	< 0.001	0.36 (0.29;0.46)	< 0.001	0.60 (0.46;0.79)	< 0.001
US vs. Canada	0.43 (0.37;0.49)	< 0.001	0.73 (0.57;0.93)	0.001	0.59 (0.45;0.79)	< 0.001	0.41 (0.26;0.62)	< 0.001	0.41 (0.32;0.51)	< 0.001	0.63 (0.50;0.79)	< 0.001	0.24 (0.19;0.31)	< 0.001	0.74 (0.56;0.97)	0.004

*Abbreviations*: *UK* United Kingdom, *US* United States, *AOR* Adjusted odds ratio, *CI* Confidence interval

^a^ Each model was adjusted for year, and sociodemographic variables including age, ethnicity, sex, perceived income adequacy, education, and the presence of a child under 18 at home

^b^ Second listed country is the reference country

In the UK, there were slightly lower odds of perceiving regular soft drinks as unhealthy in 2021, compared to 2019 and 2020 (Table 3). No other significant changes in perceived healthiness of regular soft drinks were observed over time.

**Table 3:** Perception that regular soft drinks are “unhealthy”, by country

	Australia^a^(*n* = 13 657)		Canada (*n* = 14 204)		Mexico (*n* = 14 220)		UK (*n* = 14 001)		US (*n* = 14 285)	
	AOR(99%CI)	*p*	AOR(99%CI)	*p*	AOR(99%CI)	*p*	AOR(99%CI)	*p*	AOR(99%CI)	*p*
2019 vs. 2018^b^	1.02 (0.74;1.41)	0.886	1.08 (0.78;1.49)	0.538	1.09 (0.89;1.32)	0.286	1.18 (0.88;1.57)	0.144	1.21 (0.96;1.52)	0.037
2020 vs. 2018	0.94 (0.70;1.27)	0.592	1.07 (0.78;1.48)	0.580	1.23 (1.00;1.51)	0.011	1.31 (0.99;1.73)	0.013	0.98 (0.80;1.22)	0.844
2021 vs. 2018	0.74 (0.55;1.01)	0.013	1.02 (0.73;1.43)	0.874	1.08 (0.88;1.33)	0.309	0.79 (0.61;1.02)	0.018	1.08 (0.86;1.37)	0.370
2020 vs. 2019	0.92 (0.68;1.24)	0.488	0.99 (0.73;1.35)	0.948	1.13 (0.92;1.39)	0.121	1.11 (0.82;1.51)	0.371	0.82 (0.65;1.02)	0.017
2021 vs. 2019	0.73 (0.54;0.99)	0.008	0.95 (0.68;1.31)	0.653	1.00 (0.82;1.22)	0.983	0.67 (0.50;0.89)	< 0.001	0.90 (0.71;1.14)	0.251
2021 vs. 2020	0.79 (0.60;1.05)	0.033	0.95 (0.69;1.31)	0.698	0.88 (0.71;1.09)	0.127	0.60 (0.45;0.80)	< 0.001	1.10 (0.88;1.37)	0.258
Female	2.36 (1.88;2.96)	< 0.001	1.98 (1.55;2.52)	< 0.001	1.15 (1.00;1.33)	0.011	1.96 (1.59;2.41)	< 0.001	2.06 (1.75;2.44)	< 0.001
Male [ref]										
Minority	0.82 (0.63;1.06)	0.047	0.61 (0.47;0.80)	< 0.001	0.83 (0.68;1.00)	0.011	0.80 (0.59;1.08)	0.057	0.73 (0.61;0.87)	< 0.001
Majority [ref]										
18–29 years	0.46 (0.32;0.64)	< 0.001	0.76 (0.52;1.10)	0.053	1.25 (0.92;1.71)	0.060	0.32 (0.23;0.45)	< 0.001	0.80 (0.61;1.05)	0.038
30–44 years	0.47 (0.34;0.66)	< 0.001	0.74 (0.52;1.06)	0.032	0.95 (0.70;1.30)	0.700	0.43 (0.31;0.60)	< 0.001	0.55 (0.43;0.71)	< 0.001
45–59 years	0.96 (0.67;1.38)	0.772	0.81 (0.58;1.13)	0.106	0.93 (0.67;1.27)	0.532	0.52 (0.37;0.73)	< 0.001	0.83 (0.65;1.08)	0.067
≥ 60 years [ref]										
Low education	1.09 (0.83;1.44)	0.397	0.66 (0.49;0.87)	< 0.001	0.88 (0.73;1.06)	0.086	0.75 (0.60;0.93)	0.001	0.77 (0.65;0.91)	< 0.001
Medium education	1.15 (0.89;1.49)	0.168	0.94 (0.73;1.20)	0.520	1.11 (0.88;1.39)	0.248	1.06 (0.84;1.34)	0.514	1.01 (0.83;1.23)	0.923
High education [ref]										
High income adequacy	0.87 (0.71;1.08)	0.103	1.00 (0.78;1.28)	0.999	0.88 (0.73;1.05)	0.063	0.91 (0.74;1.13)	0.275	0.74 (0.63;0.88)	< 0.001
Low income adequacy [ref]										
Children at home	0.59 (0.47;0.76)	< 0.001	0.69 (0.53;0.91)	0.001	0.96 (0.82;1.12)	0.451	0.54 (0.44;0.68)	< 0.001	0.55 (0.46;0.66)	< 0.001
No children at home [ref]										

*Abbreviations*: *UK* United Kingdom, *US* United States, *AOR* Adjusted odds ratio, *CI* Confidence interval

^a^ Each model was adjusted for year and sociodemographic variables including age, ethnicity, sex, perceived income adequacy, education, and the presence of a child under 18 at home

^b^ Second listed year is the reference year

Across all years, there was a moderate association with sex and a small association with having children at home, such that in all countries except Mexico, females and participants with no children at home had higher odds of perceiving regular soft drinks as unhealthy than males and those with children (Table 3). In Australia, the UK, and the US there was a small to moderate association with age, such that younger participants had lower odds of perceiving regular soft drinks as unhealthy than older age groups. There was also a very small association with income, ethnicity, and education such that participants with low income adequacy (US only), from an ethnic majority (Canada and US), and with high education levels (Canada, the UK and the US) had higher odds of perceiving regular soft drinks as unhealthy than those with high income adequacy, from an ethnic minority and with low education levels.

Results for perceived healthiness of other beverages can be found in supplementary files (Tables S1-S7). In Mexico, there were very small to moderate increases in the odds of perceiving 100% juice, chocolate milk, and iced tea as unhealthy in 2021 compared to 2018, and of perceiving specialty coffee as unhealthy in 2020 compared to 2018. Otherwise, trends over time were stable, except, in Australia where there was a small decrease in the odds of perceiving diet soft drinks as unhealthy between 2018 and 2021; in the US where there was a small increase in the odds of perceiving chocolate milk as unhealthy between 2018 and 2019; and in the UK where there was a very small decrease in the odds of perceiving chocolate milk as unhealthy between 2020 and 2021. In addition, mostly small to moderate differences were found by sex, level of education and presence of children at home, in some countries only. Females had higher odds of perceiving all beverages as unhealthy compared to males, participants with high education levels had higher odds of perceiving sports drinks and iced tea as unhealthy compared to less educated participants, and those with no children at home had higher odds of perceiving energy and sports drinks as unhealthy, compared to those with children at home. Small to large differences by ethnicity were also observed in some countries, such that participants from an ethnic majority had higher odds of perceiving energy drinks and specialty coffee as unhealthy compared to participants from an ethnic minority. Other differences were observed for education level, age, and presence of a child at home for some drink categories.

### Social norms

**Fig. 3:**
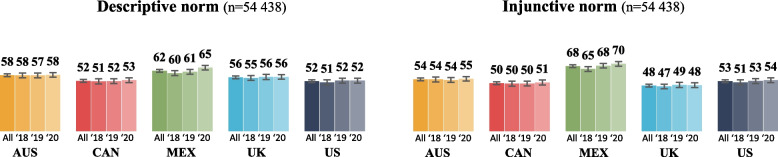
Weighted percentages of participants agreeing with the descriptive and injunctive norms^a^. Legend: ^a^ Participants were asked “People important to me TRY NOT to drink SUGARY DRINKS” for the descriptive norm and “People important to me THINK I SHOULD NOT drink SUGARY DRINKS” for the injunctive norm, with response options “agree” and “strongly agree” categorized as “agree.” Error bars represent 99% confidence intervals. Abbreviations: AUS = Australia, CAN = Canada, MEX = Mexico, UK = United-Kingdom, US = United-States, All = All countries, ‘18 = 2018, ‘19 = 2019, ‘20 = 2020

The unadjusted percentages of participants who agreed with each type of social norm discouraging sugary drink consumption are shown in Fig. 3. Agreement ranged from 52% in the US and Canada to 62% in Mexico for the descriptive norm, and from 48% in the UK to 68% in Mexico for the injunctive norm. Additional figures detailing responses to social norms are available in supplemental files (Figure S3).

There were mostly very small differences in the odds of agreeing with the descriptive norm statement between all countries, with the exceptions of no meaningful differences between the UK compared to Australia and the US compared to Canada. Overall, participants in Mexico had higher odds of agreeing with the descriptive norm compared to all other countries. Also, there were moderate differences between Mexico and the other countries for the injunctive norm, as agreement was higher in Mexico, and very small differences for all other comparisons, except for Canada compared to the UK and the US where no meaningful differences were observed (Table 4).

**Table 4:** Differences across countries in agreement with the descriptive^a^ and injunctive^b^ norms, 2018–2020 (*n* = 54 438)

	Descriptive norm^c^		Injunctive norm	
	AOR(99%CI)	*p*	AOR(99%CI)	*p*
Australia vs. Mexico^d^	0.81 (0.74;0.88)	< 0.001	0.57 (0.52;0.62)	< 0.001
Australia vs. UK	1.06 (0.98;1.15)	0.053	1.24 (1.15;1.35)	< 0.001
Australia vs. USA	1.34 (1.23;1.45)	< 0.001	1.13 (1.04;1.23)	< 0.001
Australia vs. Canada	1.26 (1.16;1.36)	< 0.001	1.16 (1.07;1.25)	< 0.001
Mexico vs. UK	1.32 (1.21;1.44)	< 0.001	2.19 (2.00;2.40)	< 0.001
Mexico vs. USA	1.66 (1.52;1.81)	< 0.001	2.00 (1.82;2.19)	< 0.001
Mexico vs. Canada	1.56 (1.42;1.70)	< 0.001	2.04 (1.86;2.24)	< 0.001
UK vs. USA	1.26 (1.16;1.37)	< 0.001	0.91 (0.84;0.99)	0.004
UK vs. Canada	1.18 (1.09;1.28)	< 0.001	0.93 (0.86;1.01)	0.027
US vs. Canada	0.94 (0.86;1.02)	0.053	1.02 (0.94;1.11)	0.515

*Abbreviations*: *UK* United Kingdom, *US* United States, *AOR* Adjusted odds ratio, *CI* Confidence interval

^a^ Participants were asked: “People important to me TRY NOT to drink SUGARY DRINKS”

^b^ Participants were asked: “People important to me THINK I SHOULD NOT drink SUGARY DRINKS”

^c^ Each model was adjusted for year, and sociodemographic variables, including age, ethnicity, sex, perceived income adequacy, education, the presence of a child under 18 at home

^d^ Second listed country is the reference country

Tables 5 and 6 show results from stratified logistic regression models examining trends over time and by sociodemographic characteristics for the descriptive and injunctive norms, separately. In Mexico, there were very small increases in the odds of agreeing with both social norm statements between 2018 and 2020, and with the descriptive norm in 2020 compared to 2019. No differences over time were found in the other countries. Except in Mexico, age and education had small to moderate associations with social norms, such that the odds of agreeing with both norms increased with age and education levels. All other meaningful differences by sociodemographic characteristics had very small associations. Males had higher odds than females of agreeing with the injunctive norm in all countries and with the descriptive norm in Mexico only. Those classified as an ethnic minority had higher odds of agreeing with the injunctive and the descriptive norms, in all countries. High perceived income adequacy was associated with greater odds of agreement with the descriptive norm in all countries except Mexico, and with the injunctive norm in Australia. Finally, participants with children at home had higher odds of agreeing with the injunctive norm than participants with no children at home in Mexico, the UK, and the US and higher odds of agreement with the descriptive norm in Mexico and the US.

**Table 5:** Agreement with the descriptive^a^ norm, by country

	Australia^b^(*n* = 10 518)		Canada (*n* = 10 714)		Mexico (*n* = 11 032)		UK (*n* = 10 972)		US (*n* = 11 202)	
	AOR(99%CI)	*p*	AOR(99%CI)	*p*	AOR(99%CI)	*P*	AOR(99%CI)	*p*	AOR(99%CI)	*p*
2019 vs. 2018^c^	1.00 (0.87;1.16)	0.955	1.02 (0.88;1.18)	0.737	1.06 (0.91;1.23)	0.314	1.04 (0.90;1.21)	0.484	1.06 (0.92;1.23)	0.291
2020 vs. 2018	1.00 (0.87;1.15)	0.950	1.05 (0.91;1.21)	0.404	1.27 (1.09;1.48)	< 0.001	1.03 (0.90;1.18)	0.561	1.04 (0.90;1.21)	0.461
2020 vs. 2019	0.99 (0.87;1.14)	0.902	1.03 (0.89;1.19)	0.610	1.20 (1.03;1.39)	0.002	0.99 (0.86;1.15)	0.879	0.98 (0.85;1.13)	0.735
Female	1.03 (0.92;1.16)	0.448	0.93 (0.83;1.05)	0.139	0.84 (0.74;0.95)	< 0.001	1.02 (0.90;1.14)	0.728	0.92 (0.82;1.04)	0.094
Male [ref]										
Minority	1.47 (1.24;1.73)	< 0.001	1.42 (1.22;1.66)	< 0.001	1.29 (1.08;1.53)	< 0.001	1.38 (1.12;1.71)	< 0.001	1.27 (1.11;1.46)	< 0.001
Majority [ref]										
18–29 years	0.54 (0.45;0.65)	< 0.001	0.54 (0.45;0.65)	< 0.001	0.30 (0.22;0.40)	< 0.001	0.40 (0.33;0.48)	< 0.001	0.60 (0.50;0.72)	< 0.001
30–44 years	0.68 (0.57;0.81)	< 0.001	0.56 (0.47;0.67)	< 0.001	0.41 (0.30;0.57)	< 0.001	0.54 (0.45;0.65)	< 0.001	0.66 (0.55;0.79)	< 0.001
45–59 years	0.77 (0.66;0.90)	< 0.001	0.69 (0.59;0.82)	< 0.001	0.63 (0.45;0.87)	< 0.001	0.57 (0.48;0.67)	< 0.001	0.69 (0.59;0.82)	< 0.001
≥60 years [ref]										
Low education	0.63 (0.54;0.73)	< 0.001	0.62 (0.53;0.72)	< 0.001	0.89 (0.76;1.05)	0.066	0.75 (0.65;0.86)	< 0.001	0.63 (0.56;0.72)	< 0.001
Medium education	0.70 (0.61;0.82)	< 0.001	0.77 (0.68;0.88)	< 0.001	0.92 (0.76;1.11)	0.242	0.79 (0.69;0.91)	< 0.001	0.80 (0.69;0.92)	< 0.001
High education [ref]										
High income adequacy	1.40 (1.24;1.58)	< 0.001	1.15 (1.02;1.30)	0.004	1.10 (0.94;1.29)	0.124	1.30 (1.15;1.47)	< 0.001	1.35 (1.19;1.53)	< 0.001
Low income adequacy[ref]										
Children at home	1.04 (0.90;1.20)	0.489	1.01 (0.87;1.17)	0.914	1.39 (1.22;1.58)	< 0.001	1.04 (0.90;1.21)	0.477	1.17 (1.01;1.37)	0.006
No children at home [ref]										

*Abbreviations:* *UK* United Kingdom, *US* United States, *AOR* Adjusted odds ratio, *CI* Confidence interval

^a^ Participants were asked: “People important to me TRY NOT to drink SUGARY DRINKS”

^b^ Each model was adjusted for year and sociodemographic variables including age, ethnicity, sex, perceived income adequacy, education, and the presence of a child under 18 at home

^c^ Second listed year is the reference year

**Table 6:** Agreement with the injunctive norm^a^, by country

	Australia^b^(*n* = 10 518)		Canada (*n* = 10 714)		Mexico (*n* = 11 032)		UK (*n* = 10 972)		US (*n* = 11 202)	
	AOR(99%CI)	*p*	AOR(99%CI)	*p*	AOR(99%CI)	*p*	AOR(99%CI)	*p*	AOR(99%CI)	*p*
2019 vs. 2018^2^	0.97 (0.85;1.12)	0.640	1.01 (0.87;1.16)	0.899	1.16 (1.00;1.36)	0.013	1.06 (0.91;1.22)	0.330	1.05 (0.90;1.21)	0.442
2020 vs. 2018	1.01 (0.88;1.16)	0.844	1.04 (0.90;1.20)	0.460	1.27 (1.09;1.49)	< 0.001	1.05 (0.91;1.20)	0.383	1.06 (0.91;1.22)	0.329
2020 vs. 2019	1.04 (0.91;1.19)	0.490	1.03 (0.90;1.19)	0.533	1.09 (0.94;1.28)	0.130	0.99 (0.86;1.15)	0.878	1.01 (0.88;1.17)	0.844
Female	0.87 (0.77;0.97)	0.001	0.84 (0.75;0.94)	< 0.001	0.76 (0.67;0.86)	< 0.001	0.82 (0.73;0.92)	< 0.001	0.83 (0.73;0.93)	< 0.001
Male [ref]										
Minority	1.47 (1.25;1.73)	< 0.001	1.39 (1.19;1.62)	< 0.001	1.36 (1.14;1.64)	< 0.001	1.52 (1.23;1.88)	< 0.001	1.39 (1.21;1.60)	< 0.001
Majority [ref]										
18–29 years	0.69 (0.58;0.82)	< 0.001	0.55 (0.46;0.66)	< 0.001	0.41 (0.30;0.56)	< 0.001	0.53 (0.44;0.64)	< 0.001	0.56 (0.47;0.67)	< 0.001
30–44 years	0.73 (0.62;0.87)	< 0.001	0.62 (0.52;0.73)	< 0.001	0.52 (0.37;0.71)	< 0.001	0.57 (0.48;0.68)	< 0.001	0.66 (0.55;0.80)	< 0.001
45–59 years	0.80 (0.68;0.93)	< 0.001	0.77 (0.66;0.91)	< 0.001	0.79 (0.57;1.09)	0.058	0.60 (0.51;0.70)	< 0.001	0.75 (0.64;0.89)	< 0.001
≥60 years [ref]										
Low education	0.70 (0.60;0.82)	< 0.001	0.77 (0.66;0.89)	< 0.001	0.97 (0.83;1.14)	0.647	0.78 (0.68;0.89)	< 0.001	0.71 (0.63;0.81)	< 0.001
Medium education	0.73 (0.63;0.85)	< 0.001	0.82 (0.73;0.93)	< 0.001	0.97 (0.80;1.19)	0.740	0.86 (0.75;0.98)	0.004	0.78 (0.68;0.90)	< 0.001
High education [ref]										
High income adequacy	1.19 (1.06;1.35)	< 0.001	0.95 (0.84;1.07)	0.247	0.97 (0.82;1.14)	0.604	1.06 (0.94;1.20)	0.179	1.04 (0.91;1.18)	0.464
Low income adequacy [ref]										
Children at home	1.11 (0.96;1.29)	0.060	1.05 (0.91;1.23)	0.360	1.35 (1.17;1.54)	< 0.001	1.17 (1.01;1.35)	0.007	1.23 (1.05;1.43)	0.001
No children at home [ref]										

*Abbreviations*: *UK* United Kingdom, *US* United States, *AOR* Adjusted odds ratio, *CI* Confidence interval

^a^ Participants were asked: “People important to me THINK I SHOULD NOT drink SUGARY DRINKS”

^b^ Each model was adjusted for year and sociodemographic variables including age, ethnicity, sex, perceived income adequacy, education, and the presence of a child under 18 at home

^c^ Second listed year is the reference year

### Association between social norms, perceived healthiness, and intake

Estimates from adjusted negative binomial regression models across all years examining the association between each correlate and beverage intake are shown in Table 7. There were mostly small but meaningful associations between perceived healthiness and intake, with slightly larger associations for most beverages in the US and for sports and diet soft drinks in most countries. Participants who perceived regular soft drinks, 100% juice, and diet soft drinks as unhealthy had lower intakes of these beverages in all countries. Except in the UK, participants who perceived sports drinks as unhealthy had lower intakes of this beverage, and participants in Australia who perceived iced tea as unhealthy had lower intakes of iced tea. For chocolate milk, meaningful differences in intake according to perceived healthiness were found only in Mexico and the US.

**Table 7:** IRR of consuming each sugary drink type (for perceived healthiness) and any sugary drink (for social norms) in the past 7 days

		Australia^a^		Canada		Mexico		UK		US	
		IRR(99%CI)	*p*	IRR(99%CI)	*p*	IRR(99%CI)	*p*	IRR(99%CI)	*p*	IRR(99%CI)	*p*
**Perceived healthiness**^**b**^(ref = not unhealthy)	Regular soft drinks (*n* = 70 367)	0.55 (0.46;0.65)	< 0.001	0.55 (0.46;0.65)	< 0.001	0.77 (0.73;0.82)	< 0.001	0.58 (0.49;0.69)	< 0.001	0.77 (0.69;0.86)	< 0.001
	100% juice (*n* = 8577)	0.58 (0.43;0.78)	< 0.001	0.44 (0.34;0.57)	< 0.001	0.72 (0.60;0.86)	< 0.001	0.52 (0.39;0.68)	< 0.001	0.47 (0.35;0.63)	< 0.001
	Diet soft drinks (*n* = 8704)	0.39 (0.23;0.67)	< 0.001	0.36 (0.21;0.61)	< 0.001	0.43 (0.28;0.68)	< 0.001	0.41 (0.28;0.60)	< 0.001	0.37 (0.25;0.56)	< 0.001
	Energy drinks (*n* = 8406)	0.67 (0.19;2.41)	0.423	0.33 (0.07;1.66)	0.077	0.59 (0.29;1.21)	0.058	1.10 (0.26;4.65)	0.862	0.39 (0.17;0.92)	0.005
	Sports drinks (*n* = 8009)	0.27 (0.13;0.54)	< 0.001	0.38 (0.18;0.80)	0.001	0.46 (0.34;0.64)	< 0.001	0.64 (0.29;1.40)	0.144	0.37 (0.23;0.60)	< 0.001
	Chocolate milk (*n* = 8048)	0.71 (0.39;1.29)	0.143	0.79 (0.48;1.30)	0.219	0.57 (0.39;0.83)	< 0.001	1.36 (0.70;2.64)	0.234	0.44 (0.25;0.78)	< 0.001
	Iced tea (*n* = 8094)	0.33 (0.17;0.62)	< 0.001	0.67 (0.38;1.20)	0.079	1.03 (0.75;1.42)	0.796	0.96 (0.47;1.95)	0.871	0.78 (0.48;1.26)	0.183
**Social norm**^**c**^ (ref = not agree)	Descriptive norm (*n* = 54 438)	0.73 (0.67;0.80)	< 0.001	0.77 (0.71;0.84)	< 0.001	0.91 (0.87;0.96)	< 0.001	0.81 (0.74;0.89)	< 0.001	0.86 (0.80;0.92)	< 0.001
	Injunctive norm (*n* = 54 438)	0.84 (0.77;0.92)	< 0.001	0.95 (0.88;1.04)	0.142	0.95 (0.90;1.00)	0.007	0.90 (0.82;0.99)	0.005	0.95 (0.88;1.02)	0.047

*Abbreviations*: *UK* United Kingdom, *US* United States, *IRR* Incident risk ratio, *CI* Confidence interval

^a^ Each model was adjusted for year and sociodemographic variables including age, ethnicity, sex, perceived income adequacy, education, and the presence of a child under 18 at home

^b^ These models examined the association between perceived healthiness toward each drink category and the intake of each drink category in the past 7 days

^c^ These models examined the association between social norms and the intake of any sugary drink in the past 7 days

Social norms had mostly very small associations with sugary drink intake. Agreement with the descriptive norm was associated with lower intake of sugary drinks in all countries, and agreement with the injunctive norm was associated with lower intake in Australia, Mexico, and the UK.

## Discussion

This study examined correlates of sugary drink consumption using a repeat cross-sectional study design and identified differences between countries and over time. Overall, participants in Australia had greater odds of perceiving most sugary drinks as unhealthy, while participants in the US and Mexico had lower odds of perceiving them as unhealthy, compared to other countries. Over time, participants in Mexico had greater odds of perceiving 100% juice, chocolate milk, iced tea, and specialty coffee as unhealthy. In addition, agreement with the descriptive and the injunctive norms was higher in Mexico, than in other countries, and increased over time in Mexico only. Some differences were observed based on sociodemographic characteristics, including male participants having lower odds of perceiving sugary drinks as unhealthy, and females, younger individuals, those from an ethnic majority, and those with lower levels of education having lower odds of agreeing with both social norms examined. Our results also showed that perceiving a beverage as unhealthy and agreeing with social norms discouraging sugary drink consumption were generally associated with lower odds of consuming sugary drinks.

### Perceived healthiness

Our findings reflect existing evidence that energy drinks, regular and diet soft drinks are widely perceived as unhealthy, while 100% juice and water are less likely to be perceived as unhealthy [36–38], and that 100% juice is perceived as healthier than regular soft drinks [45]. These findings may be attributed to the nutritional composition of these beverages, as consumers often perceive high sugar or high fructose corn syrup content, caffeine, and artificial sweeteners as concerning [36, 46], whereas they perceive fruit content and vitamins as positive attributes [36, 46, 47]. The perception of 100% juice may also reflect less consistent evidence on the health effects of this beverage [48, 49]. Indeed, 100% juices are still considered a serving of fruit in some, but not all, dietary guidelines [50], and are not consistently included in public health nutrition policies [51, 52], making them appear healthier to some consumers.

In Mexico, more participants perceived 100% juice, chocolate milk, specialty coffee, and iced tea, as unhealthy over time, which is in line with the implementation of the mandatory FOPs warning labels in 2020 (i.e., prominent stop signs on packaging to identify unhealthy beverages and warn consumers on the presence of caffeine and non-caloric sweeteners) [41]. Indeed, FOP labeling, and specifically “high in” symbols, has been shown to alter consumers' health perceptions of sugary drinks [53]. This suggests that perceptions are modifiable and highlights the potential for public health policy to influence beliefs.

Participants in the UK were less likely to perceive regular soft drinks as unhealthy in 2021 compared to 2019. Negative attitudes towards sugary drinks may have been heightened in the UK in 2018 as a result of the implementation of the soft drinks industry levy, a “tax” that was applied to manufacturers according to the amount of sugar in beverages to encourage reformulation and sugar reductions in beverages [54]. It is also possible that the reduction in the amount of sugar in beverages following the levy [55] may have led UK participants to perceive these drinks as “healthier”.

Our results also show differences in perceived healthiness across countries and sociodemographic groups. For example, higher proportions of participants in Australia perceived most beverages as unhealthy, which may reflect the country’s recent efforts to raise awareness of the negative effects of sugary drinks, such as the Live Lighter® and Rethink Sugary Drink campaigns [56, 57]. In addition, female participants, and those with high education levels tended to have higher odds of perceiving some sugary drinks as unhealthy. Previous research has indeed shown that women and those with higher education levels have greater nutrition knowledge [58] and identify health as a more important factor in food choices [59], which might explain these results. Perceptions also differed between adults with and without children at home. The strong preference of children for sugary drinks [60], the various marketing practices for children’s drinks aimed at parents [61], and the fact that parents are more exposed to sugary drinks through their children could result in parental confusion, potentially causing misperceptions about the healthfulness of sugary drinks. However, given that the data are cross-sectional, the direction of this association is unclear. Differences could also be explained by cognitive dissonance, such that parents who are more exposed to sugary drinks or who purchase more sugary drinks for their children change their perceived healthiness of these beverages to reduce feelings of discomfort or guilt.

Overall, our results show associations between perceiving a beverage as unhealthy and consuming less of that beverage, consistent with other studies [38, 62]. Associations were somewhat less prevalent for more “ambiguous” drinks (e.g., drinks that may be perceived as healthier due to some properties or marketing, but are in fact high in added sugar, such as sports drinks, chocolate milk, and iced tea) in the UK. This may in part be due to differences in food environments such as the marketing, price, and availability of these drinks in the UK.

The findings suggest that policies that influence perceptions of sugary drinks and target specific subgroups of the population could contribute to efforts to reduce sugary drink consumption. For instance, it may be of interest to target parental beliefs about sugary drinks, such as through targeted education campaigns, as parental misconceptions may influence which drinks they buy for their children [36, 63]. Sex-specific campaigns may also be relevant as males were less likely to perceive sugary drinks as unhealthy. Our findings may help inform which beverage categories should be included in regulations or policies, given, for example, that the consumption of sports drinks and chocolate milk is increasing [15, 17] and that they were perceived as “healthier” products in our study compared to products with similar levels of sugars. Our results also highlight the opportunity to better regulate food marketing practices that are likely to lead to misperceptions of healthiness, such as nutrition claims on beverages [64] or other persuasive advertising strategies commonly used in the marketing of sports, energy, and fruit drinks [65, 66].

### Social norms

Approximately half of the sample agreed with the descriptive and injunctive norms indicating perceptions of negative attitudes toward sugary drinks in their social circles, with higher agreement in Mexico. This contrasts with previous studies published in 2011, and 2020 which showed norms in favor of SSB consumption in Mexico and Latino communities in the US [67–69]. Greater agreement may be partly explained by Mexico's more collectivist beliefs, with higher support of government policies and stronger negative reactions to norm violators than individualist countries [70, 71]. In the current study, agreement with each social norm in Mexico was higher in 2020 than in 2018, which may be related to the many recent governmental and non-governmental efforts to reduce the consumption of sugary drinks and raise awareness among the population, including governmental actions to improve food environments and major mobilizations of civil society organizations to promote public education campaigns on the health effects of sugary drinks and SSBs and generate support for public policies that address this issue [72–74]. These may have contributed to a change in norms in this country, especially as consumers in Mexico are known to be more aware of public campaigns to promote healthy eating than in other countries [75]. Differences in sociodemographic characteristics have also been found in other research examining social norms towards SSBs. For example, in our study, younger participants, especially those between 18 and 29 years old, were less likely to agree with both norms, consistent with another study on perceived societal disapproval of SSBs [34]. This could be explained by the role that SSBs play in young adults’ social interactions and social settings [76, 77], along with a change in the social acceptability of these drinks over time [78].

Despite higher agreement with both norms in Mexico, there was little association between norms and sugary drink intake in this country. The reduced association between social norms and behaviour may be related to strong cultural ties and historically high consumption of SSB in Mexico, which may weaken the link between beliefs and behaviors [67, 69, 79] or to the industry's strategies to counteract governmental efforts [80]. In all countries, sugary drink intake was more strongly associated with the descriptive norm compared to the injunctive norm, in line with previous studies suggesting that injunctive norms may be more closely related to the intention to engage in a behavior, whereas the descriptive norm is directly related to the behavior [28, 34]. Correcting misperceptions about descriptive norms by addressing how people perceive others’ sugary drink intake may therefore hold promise for reduction efforts [81]. More broadly, denormalizing sugary drink consumption, a technique that has been effective for smoking [82], may be particularly relevant for young people with lower education, as they were less likely to agree with both norms in our study and as their consumption is higher than older and more educated groups [83].

### Strengths and limitations

This study has several strengths. First, the study used a large sample size, the same measures in five countries over four years, and allowed an in-depth examination of sociodemographic differences that may help identify which populations may benefit most from interventions or special consideration in population health approaches. In addition, a wide range of beverage categories that have rarely been examined individually were included, which may help inform policy decisions related to specific beverage categories. Moreover, this study will serve as a baseline for assessing the impact of future policies, such as FOP labeling, which will be introduced in Canada in 2026 [84], and is under consideration in the US [85].

This study is subject to limitations common to survey research. Participants were recruited using non-probability-based sampling and the results do not necessarily provide nationally representative estimates; however, the analyses were weighted to better represent the populations in each country. Moreover, participants with higher education were overrepresented in Mexico compared to other countries, suggesting the need to conduct research with populations with lower levels of education to confirm that the pattern of the results applies to them. However, the null association between education and perceived healthiness and social norms in Mexico suggests that selection bias may not have compromised the overall results. Also, the cross-sectional nature of these data does not allow for the establishment of causal relationships. The use of binary variables for social norms and perceived healthiness, as opposed to the 5- and 7-point continuous Likert scales, resulted in a loss of information. However, sensitivity analyses conducted using continuous variables based on all Likert scale levels showed similar patterns of results (data not shown), and figures showing the distribution of the response from the Likert scale level are provided in the Supplementary Figure S2 and S3.

Despite its previous evaluation for validity [42], the use of the self-reported Beverage Frequency Questionnaire is subject to measurement error, which may vary between different groups and over time as beverage markets are changing. Indeed, consumption of new beverage categories may not have been captured by this questionnaire, resulting in an underestimation of consumption, and the exclusion of coffee with sugar and specialty coffee in the sugary drink intake variable may have led to an underestimation of the prevalence of consumers, particularly in Australia, where ready-to-drink coffee is more commonly consumed [86]. Also, the Beverage Frequency Questionnaire reflects consumption over the past 7 days, which may improve the ability to assess consumption of less frequently consumed beverages, but also increases the difficulty of recall.

For the perceived healthiness variable, FOP “excess” warning labels were added to the sugary drink images of the Mexico questionnaire in 2020, in line with national regulations and to represent the current food environment in Mexico, which may have affected participants' perceptions. Nevertheless, the images shown in the survey were representative of the "real world" environment in which the participants are exposed to sugary drinks. This measure also only assessed one brand of beverages, and perceptions or actual healthiness may vary between various brands. Moreover, as the term "sugary drink" is more common, it was used in the social norm questions. However, the definition provided to participants prior to answering these questions referred to SSBs (containing added sugars), rather than sugary drinks (containing free sugars). Participants may have answered differently if the definition provided included 100% juice. This limitation highlights the critical need for clear, standardized definitions and nutrient profiling systems to accurately distinguish between “sugar”, “sweetened” and “sugar-sweetened' beverages”, particularly in policy discussions aimed at regulating these products [87]. Finally, the COVID-19 pandemic may have had an impact on participants’ responses, particularly on perceived social norms, as social interactions and social relationships changed significantly in 2020, although the directionality of this potential impact is unclear [88].

## Conclusion

This study showed that perceived healthiness and social norms related to sugary drinks vary across countries and sociodemographic characteristics. Our findings highlighted several sugary drink categories, such as 100% juice, chocolate milk, and sports drinks, which are generally perceived as healthier by consumers and therefore warrant policy attention. In most cases, perceiving a beverage as unhealthy and agreeing with social norms discouraging sugary drink intake were associated with lower sugary drink consumption. This highlights the importance of policies that can influence perceptions of sugary drinks and cultural attitudes to ultimately contribute to a reduction in sugary drink consumption. Shifts in perceived healthiness and social norms observed in Mexico, a country actively addressing the issue of sugary drinks, suggest that these correlates are not fixed and that policies can play a role in shaping beliefs about these beverages. 

## Supplementary Information

Additional files 1. Figure S1: Images shown to participants for questions on perceived healthiness, by country. Figure S2: Weighted percentages of participants reporting each level of the perceived healthiness Likert scale by beverage category, across all countries and years. Figure S3: Weighted percentages of participants reporting each level of the Likert scale for the descriptive and injunctive norms, across all countries and years.

Additional files 2. Tables S1 to S7: Results for perceived healthiness of other beverages.

## Data Availability

Data are available directly from the International Food Policy Study team on reasonable request (see: foodpolicystudy.com).

## Abbreviations

AOR: Adjusted odds ratio
FOP: Front-of-pack labeling
IFPS: International Food Policy Study
IRR: Incident risk ratio
SSB: Sugar-sweetened beverage
UK: United Kingdom
US: United-States
