# Supplementary material for: Perceived healthiness of sugary drinks and related social norms among adults in five countries: evidence from the International Food Policy Study
Source: Nutr J. 2025 Jan 29;24:19. doi: 10.1186/s12937-024-01063-8 (PMC11776124; doi:10.1186/s12937-024-01063-8)
Supplement: Supplementary file 1 — Additional files 1. Figure S1: Images shown to participants for questions on perceived healthiness, by country. Figure S2: Weighted percentages of participants reporting each level of the perceived healthiness Likert scale by beverage category, across all countries and years. Figure S3: Weighted percentages of participants reporting each level of the Likert scale for the descriptive and injunctive norms, across all countries and years. [file 12937_2024_1063_MOESM1_ESM.docx]

**Figure S1**: Images shown to participants for questions on perceived healthiness, by country

| Australia | 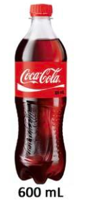 | 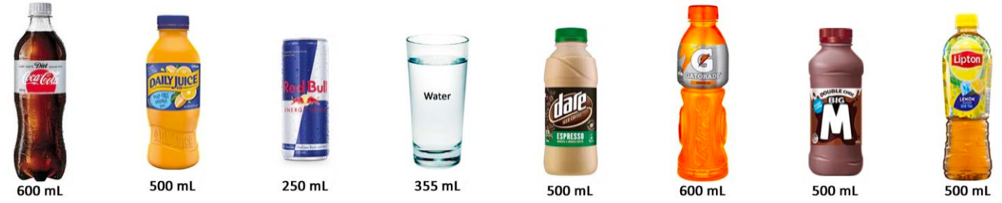 |
| --- | --- | --- |
| Canada | 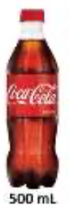 | 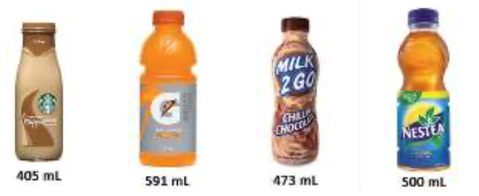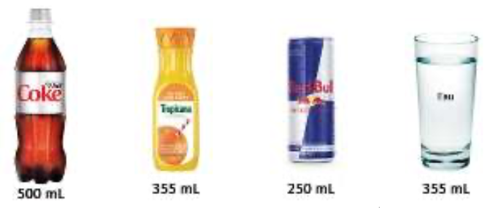 |
| Mexico* | 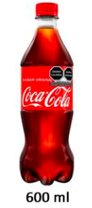 | 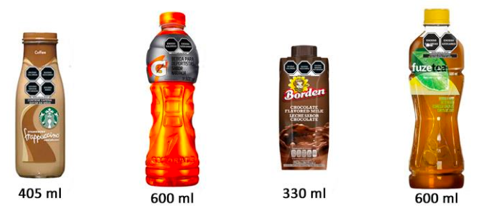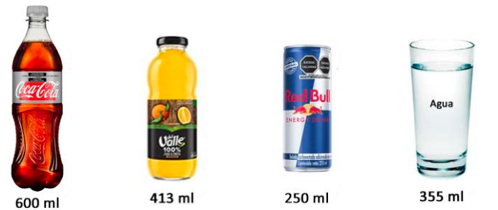 |
| UK | 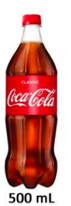 | 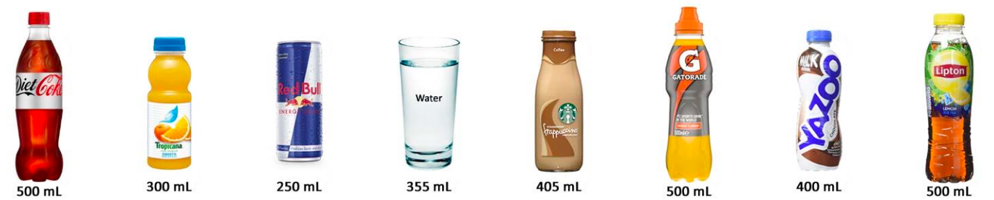 |
| US | 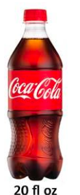 | 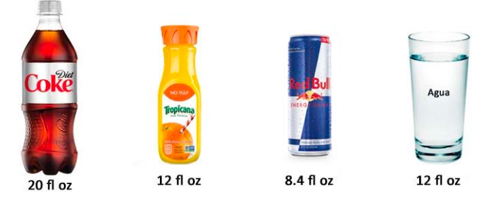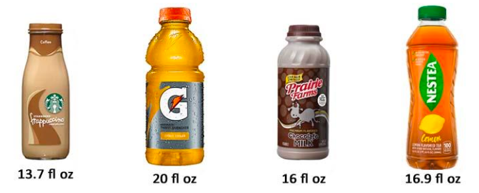 |

Abbreviations: UK = United Kingdom; US=United States;

Note: In 2020, FOP “high in” symbols were added in the Mexico questionnaire, in line with national regulations

**Figure S2**: Weighted percentages of participants reporting each level of the perceived healthiness Likert scale by beverage category, across all countries and years


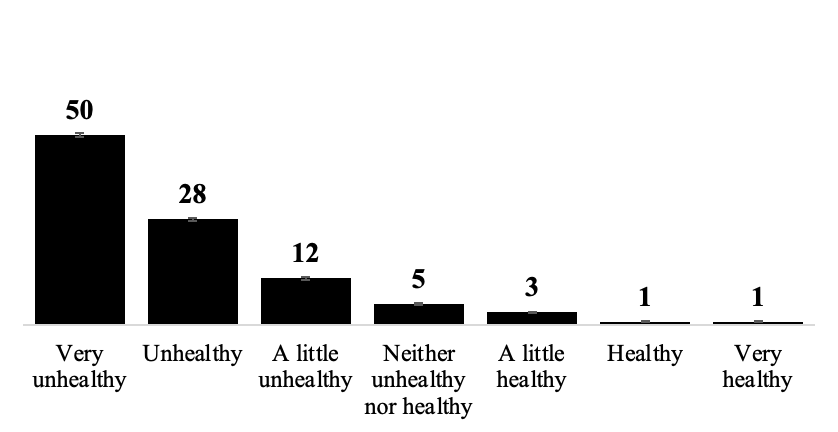

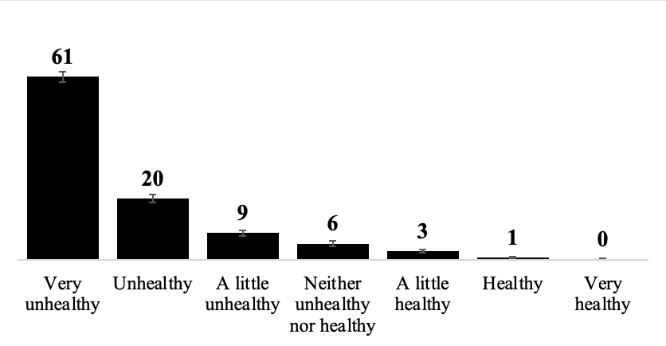

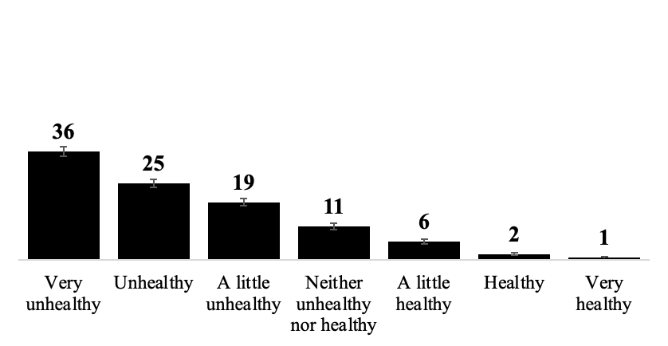

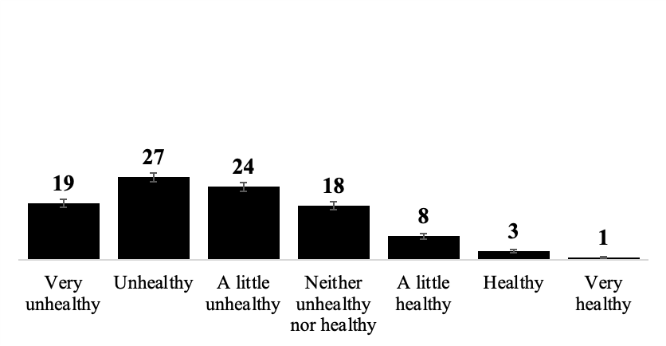


**Specialty coffee** *(n=8193)*

**Energy drinks** *(n=8406)*

**Regular soft drinks** *(n=70 367)*

**Diet soft drinks** *(n=8704)*


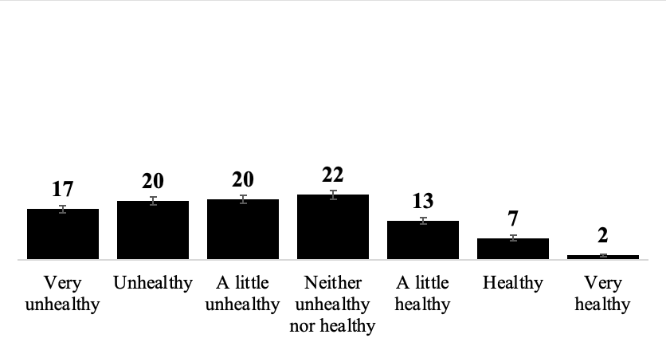

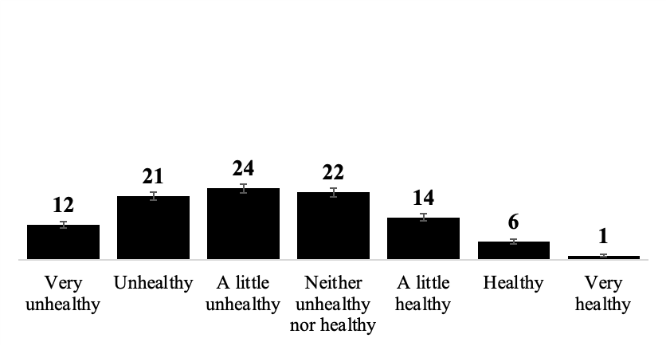


**Iced tea** *(n=8094)*

**Sports drinks** *(n=8009)*


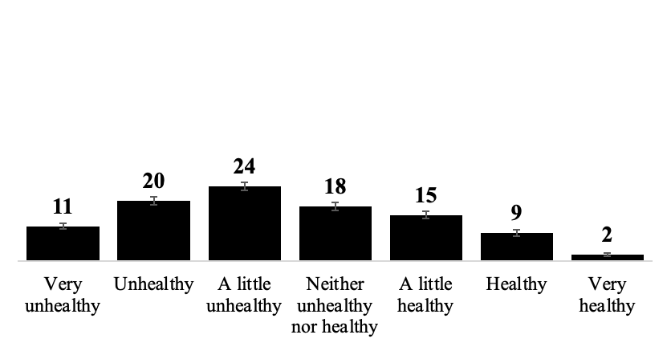

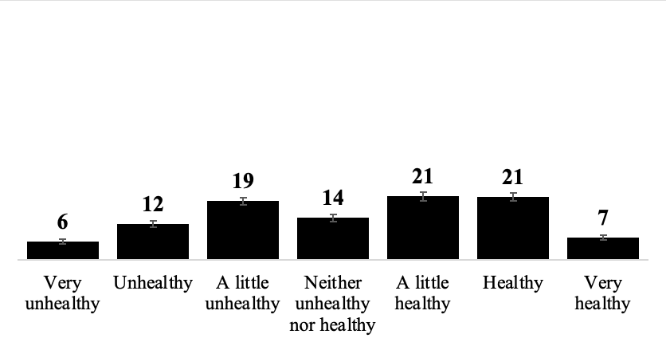


**Chocolate milk** *(n=8048)*

**100% juice** *(n=8577)*


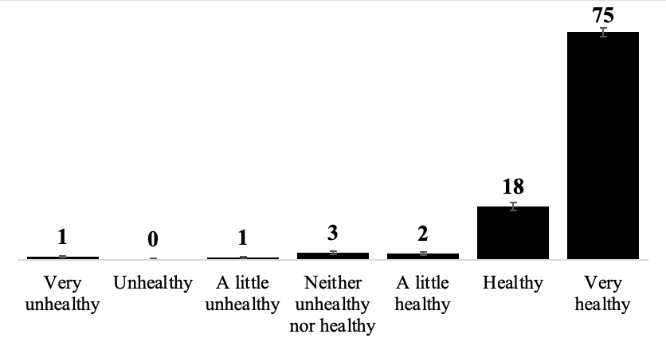


**Water** *(n=8274)*

Legend: Error bars represent 99% confidence intervals.

**Figure S3**: Weighted percentages of participants reporting each level of the Likert scale for the descriptive and injunctive norms, across all countries and years.


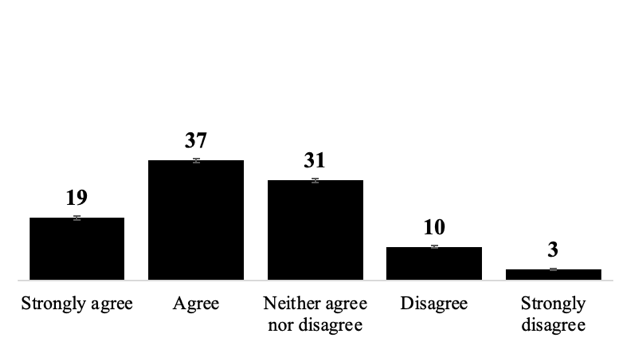


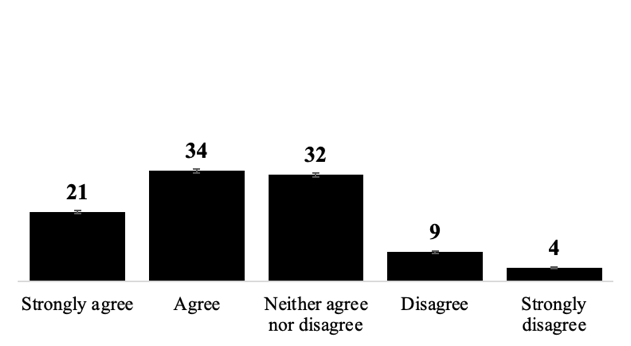


**Descriptive norm :** “People important to me TRY NOT to drink SUGARY DRINKS” *(n=54 438)*

**Injunctive norm:** “People important to me THINK I SHOULD NOT drink SUGARY DRINKS” *(n=54 438)*

Legend: Error bars represent 99% confidence intervals.
