# Supplementary material for: Perceived healthiness of sugary drinks and related social norms among adults in five countries: evidence from the International Food Policy Study
Source: Nutr J. 2025 Jan 29;24:19. doi: 10.1186/s12937-024-01063-8 (PMC11776124; doi:10.1186/s12937-024-01063-8)
Supplement: Supplementary file 2 — Additional files 2. Tables S1 to S7: Results for perceived healthiness of other beverages. [file 12937_2024_1063_MOESM2_ESM.docx]

**Table S1**: Perception that 100% juice is “unhealthy”, by country

|  | Australia^a^  (n=1636) | | Canada  (n=1759) | | Mexico  (n=1664) | | UK  (n=1789) | | US  (n=1729) | |
| --- | --- | --- | --- | --- | --- | --- | --- | --- | --- | --- |
|  | AOR(99%CI) | *p* | AOR(99%CI) | *p* | AOR(99%CI) | *p* | AOR(99%CI) | *p* | AOR(99%CI) | *p* |
| 2019 vs 2018^b^ | 1.00 (0.67;1.50) | 0.989 | 1.02 (0.65;1.60) | 0.905 | 1.27 (0.81;1.99) | 0.173 | 1.30 (0.85;1.98) | 0.110 | 1.08 (0.68;1.74) | 0.661 |
| 2020 vs 2018 | 0.96 (0.64;1.44) | 0.796 | 1.21 (0.76;1.93) | 0.297 | 2.12 (1.37;3.28) | <0.001 | 0.92 (0.61;1.38) | 0.594 | 0.76 (0.45;1.27) | 0.162 |
| 2021 vs 2018 | 0.83 (0.55;1.25) | 0.238 | 0.90 (0.57;1.42) | 0.549 | 1.71 (1.10;2.64) | 0.002 | 1.12 (0.73;1.71) | 0.500 | 0.92 (0.56;1.50) | 0.646 |
| 2020 vs 2019 | 0.96 (0.64;1.43) | 0.784 | 1.18 (0.74;1.89) | 0.352 | 1.67 (1.06;2.63) | 0.004 | 0.71 (0.46;1.10) | 0.041 | 0.70 (0.41;1.18) | 0.078 |
| 2021 vs 2019 | 0.83 (0.55;1.24) | 0.230 | 0.88 (0.56;1.40) | 0.476 | 1.34 (0.85;2.12) | 0.094 | 0.86 (0.55;1.35) | 0.392 | 0.85 (0.51;1.40) | 0.393 |
| 2021 vs 2020 | 0.86 (0.58;1.30) | 0.354 | 0.74 (0.46;1.19) | 0.106 | 0.81 (0.52;1.25) | 0.208 | 1.22 (0.78;1.89) | 0.254 | 1.21 (0.70;2.08) | 0.364 |
| Female | 1.20 (0.90;1.60) | 0.102 | 1.52 (1.10;2.10) | 0.001 | 1.48 (1.08;2.02) | 0.001 | 1.68 (1.23;2.28) | <0.001 | 1.66 (1.14;2.43) | 0.001 |
| Male [ref] |  |  |  |  |  |  |  |  |  |  |
| Minority | 0.83 (0.56;1.24) | 0.228 | 1.12 (0.71;1.75) | 0.527 | 0.95 (0.62;1.46) | 0.767 | 0.85 (0.45;1.60) | 0.510 | 1.22 (0.80;1.87) | 0.228 |
| Majority [ref] |  |  |  |  |  |  |  |  |  |  |
| 18-29 years | 1.29 (0.84;1.97) | 0.131 | 1.26 (0.75;2.11) | 0.244 | 2.21 (1.15;4.24) | 0.002 | 0.45 (0.28;0.73) | <0.001 | 1.08 (0.61;1.90) | 0.730 |
| 30-44 years | 1.32 (0.86;2.01) | 0.097 | 1.59 (0.97;2.61) | 0.015 | 1.38 (0.70;2.74) | 0.222 | 0.83 (0.51;1.34) | 0.310 | 1.28 (0.75;2.20) | 0.235 |
| 45-59 years | 1.09 (0.74;1.62) | 0.555 | 1.05 (0.67;1.65) | 0.766 | 1.24 (0.62;2.47) | 0.433 | 1.01 (0.67;1.51) | 0.959 | 1.21 (0.73;1.98) | 0.331 |
| ≥60 years [ref] |  |  |  |  |  |  |  |  |  |  |
| Low education | 0.71 (0.48;1.03) | 0.019 | 0.39 (0.26;0.60) | <0.001 | 0.97 (0.64;1.46) | 0.847 | 0.71 (0.50;1.01) | 0.013 | 0.78 (0.53;1.16) | 0.103 |
| Medium education | 0.89 (0.62;1.29) | 0.434 | 0.53 (0.37;0.74) | <0.001 | 0.80 (0.50;1.28) | 0.220 | 0.71 (0.50;1.01) | 0.012 | 0.85 (0.54;1.33) | 0.338 |
| High education [ref] |  |  |  |  |  |  |  |  |  |  |
| High income adequacy | 1.00 (0.75;1.35) | 0.979 | 0.94 (0.68;1.31) | 0.643 | 1.00 (0.65;1.52) | 0.976 | 1.01 (0.73;1.40) | 0.930 | 1.05 (0.71;1.54) | 0.758 |
| Low income adequacy [ref] |  |  |  |  |  |  |  |  |  |  |
| Children at home | 0.84 (0.58;1.21) | 0.206 | 0.71 (0.47;1.08) | 0.035 | 0.75 (0.53;1.05) | 0.025 | 0.79 (0.52;1.21) | 0.158 | 0.63 (0.40;1.01) | 0.011 |
| No children at home [ref] |  |  |  |  |  |  |  |  |  |  |

Abbreviations: UK = United Kingdom; US=United States; AOR= Adjusted odds ratio; CI=Confidence interval.

^a^ Each model was adjusted for year and sociodemographic variables including age, ethnicity, sex, perceived income adequacy, education, and the presence of a child under 18 at home.

^b^ Second listed year is the reference year.

**Table S2** Perception that diet soft drinks are “unhealthy”, by country

|  | Australia^a^  (n=1641) | | Canada  (n=1765) | | Mexico  (n=1778) | | UK  (n=1803) | | US  (n=1717) | |
| --- | --- | --- | --- | --- | --- | --- | --- | --- | --- | --- |
|  | AOR(99%CI) | *p* | AOR(99%CI) | *p* | AOR(99%CI) | *p* | AOR(99%CI) | *p* | AOR(99%CI) | *p* |
| 2019 vs 2018^b^ | 1.00 (0.55;1.83) | 0.985 | 0.92 (0.50;1.72) | 0.744 | 1.17 (0.69;1.99) | 0.436 | 1.08 (0.66;1.75) | 0.688 | 0.92 (0.54;1.58) | 0.706 |
| 2020 vs 2018 | 0.87 (0.49;1.55) | 0.526 | 0.87 (0.47;1.62) | 0.564 | 0.87 (0.51;1.49) | 0.517 | 1.17 (0.74;1.85) | 0.386 | 0.74 (0.44;1.26) | 0.148 |
| 2021 vs 2018 | 0.54 (0.31;0.94) | 0.004 | 0.87 (0.47;1.60) | 0.544 | 1.23 (0.72;2.11) | 0.321 | 0.98 (0.63;1.55) | 0.926 | 0.73 (0.44;1.21) | 0.107 |
| 2020 vs 2019 | 0.86 (0.48;1.55) | 0.520 | 0.94 (0.51;1.72) | 0.796 | 0.75 (0.44;1.27) | 0.155 | 1.08 (0.65;1.80) | 0.688 | 0.80 (0.47;1.37) | 0.289 |
| 2021 vs 2019 | 0.54 (0.30;0.95) | 0.005 | 0.94 (0.52;1.70) | 0.774 | 1.05 (0.61;1.80) | 0.820 | 0.91 (0.55;1.50) | 0.635 | 0.79 (0.47;1.31) | 0.228 |
| 2021 vs 2020 | 0.62 (0.36;1.08) | 0.026 | 0.99 (0.55;1.80) | 0.980 | 1.41 (0.83;2.40) | 0.097 | 0.84 (0.52;1.35) | 0.353 | 0.98 (0.59;1.63) | 0.919 |
| Female | 1.45 (0.98;2.15) | 0.015 | 2.17 (1.38;3.41) | <0.001 | 1.27 (0.86;1.86) | 0.113 | 1.47 (1.05;2.07) | 0.003 | 2.22 (1.52;3.26) | <0.001 |
| Male [ref] |  |  |  |  |  |  |  |  |  |  |
| Minority | 0.92 (0.54;1.56) | 0.673 | 0.61 (0.35;1.07) | 0.024 | 0.86 (0.53;1.39) | 0.406 | 1.18 (0.64;2.18) | 0.496 | 0.79 (0.53;1.20) | 0.146 |
| Majority [ref] |  |  |  |  |  |  |  |  |  |  |
| 18-29 years | 0.64 (0.35;1.15) | 0.049 | 1.16 (0.58;2.31) | 0.584 | 1.23 (0.54;2.83) | 0.522 | 0.82 (0.48;1.40) | 0.332 | 1.22 (0.69;2.18) | 0.372 |
| 30-44 years | 0.87 (0.49;1.56) | 0.545 | 0.85 (0.43;1.67) | 0.537 | 1.17 (0.51;2.69) | 0.635 | 0.85 (0.50;1.43) | 0.419 | 0.67 (0.38;1.20) | 0.079 |
| 45-59 years | 0.81 (0.46;1.43) | 0.344 | 1.06 (0.58;1.92) | 0.814 | 1.08 (0.46;2.55) | 0.816 | 0.78 (0.48;1.26) | 0.185 | 0.94 (0.56;1.57) | 0.747 |
| ≥60 years [ref] |  |  |  |  |  |  |  |  |  |  |
| Low education | 1.15 (0.68;1.97) | 0.487 | 0.58 (0.33;1.02) | 0.013 | 0.82 (0.50;1.34) | 0.291 | 0.59 (0.40;0.86) | <0.001 | 0.99 (0.66;1.49) | 0.966 |
| Medium education | 1.05 (0.64;1.73) | 0.805 | 0.65 (0.39;1.08) | 0.027 | 1.34 (0.70;2.55) | 0.250 | 0.69 (0.47;1.03) | 0.016 | 0.90 (0.58;1.41) | 0.561 |
| High education [ref] |  |  |  |  |  |  |  |  |  |  |
| High income adequacy | 0.79 (0.52;1.21) | 0.154 | 1.08 (0.68;1.71) | 0.663 | 0.75 (0.47;1.18) | 0.101 | 0.79 (0.56;1.13) | 0.089 | 0.83 (0.55;1.24) | 0.229 |
| Low income adequacy [ref] |  |  |  |  |  |  |  |  |  |  |
| Children at home | 0.74 (0.45;1.22) | 0.123 | 0.99 (0.53;1.82) | 0.950 | 0.86 (0.57;1.30) | 0.343 | 0.71 (0.47;1.08) | 0.035 | 0.69 (0.43;1.09) | 0.035 |
| No children at home [ref] |  |  |  |  |  |  |  |  |  |  |

Abbreviations: UK = United Kingdom; US=United States; AOR= Adjusted odds ratio; CI=Confidence interval.

^a^ Each model was adjusted for year and sociodemographic variables including age, ethnicity, sex, perceived income adequacy, education, and the presence of a child under 18 at home.

^b^ Second listed year is the reference year.

**Table S3**: Perception that regular energy drinks are “unhealthy”, by country

|  | Australia^a^  (n=1618) | | Canada  (n=1702) | | Mexico  (n=1672) | | UK  (n=1728) | | US  (n=1686) | |
| --- | --- | --- | --- | --- | --- | --- | --- | --- | --- | --- |
|  | AOR(99%CI) | *p* | AOR(99%CI) | *p* | AOR(99%CI) | *p* | AOR(99%CI) | *p* | AOR(99%CI) | *p* |
| 2019 vs 2018^b^ | 1.03 (0.38;2.76) | 0.941 | 0.66 (0.24;1.84) | 0.299 | 1.09 (0.60;1.97) | 0.708 | 0.89 (0.41;1.93) | 0.699 | 0.96 (0.48;1.89) | 0.869 |
| 2020 vs 2018 | 0.63 (0.25;1.58) | 0.199 | 1.35 (0.46;4.00) | 0.471 | 1.08 (0.56;2.10) | 0.757 | 1.83 (0.80;4.17) | 0.060 | 0.80 (0.44;1.46) | 0.340 |
| 2021 vs 2018 | 0.47 (0.19;1.15) | 0.031 | 1.22 (0.46;3.22) | 0.605 | 1.06 (0.58;1.93) | 0.814 | 1.11 (0.53;2.32) | 0.724 | 0.93 (0.48;1.81) | 0.780 |
| 2020 vs 2019 | 0.62 (0.26;1.45) | 0.145 | 2.04 (0.74;5.62) | 0.070 | 0.99 (0.52;1.89) | 0.980 | 2.05 (0.85;4.97) | 0.036 | 0.84 (0.44;1.59) | 0.473 |
| 2021 vs 2019 | 0.46 (0.20;1.05) | 0.016 | 1.83 (0.71;4.71) | 0.100 | 0.97 (0.54;1.72) | 0.889 | 1.24 (0.55;2.79) | 0.489 | 0.97 (0.48;1.95) | 0.917 |
| 2021 vs 2020 | 0.75 (0.35;1.60) | 0.327 | 0.90 (0.34;2.38) | 0.775 | 0.98 (0.51;1.88) | 0.922 | 0.61 (0.26;1.39) | 0.119 | 1.16 (0.64;2.13) | 0.520 |
| Female | 2.16 (1.15;4.04) | 0.002 | 2.74 (1.28;5.83) | 0.001 | 1.37 (0.86;2.18) | 0.083 | 2.24 (1.24;4.06) | <0.001 | 2.32 (1.40;3.83) | <0.001 |
| Male [ref] |  |  |  |  |  |  |  |  |  |  |
| Minority | 0.30 (0.16;0.58) | <0.001 | 0.41 (0.20;0.85) | 0.002 | 0.82 (0.47;1.46) | 0.382 | 0.33 (0.15;0.73) | <0.001 | 0.53 (0.32;0.86) | 0.001 |
| Majority [ref] |  |  |  |  |  |  |  |  |  |  |
| 18-29 years | 0.80 (0.32;2.02) | 0.540 | 1.95 (0.67;5.65) | 0.107 | 1.09 (0.42;2.83) | 0.824 | 0.71 (0.28;1.79) | 0.335 | 0.91 (0.42;1.96) | 0.742 |
| 30-44 years | 0.65 (0.26;1.62) | 0.220 | 0.71 (0.27;1.84) | 0.355 | 1.10 (0.40;2.99) | 0.807 | 0.99 (0.43;2.25) | 0.972 | 0.53 (0.26;1.07) | 0.020 |
| 45-59 years | 1.31 (0.49;3.47) | 0.482 | 1.16 (0.38;3.50) | 0.734 | 1.10 (0.39;3.09) | 0.815 | 0.87 (0.37;2.06) | 0.677 | 0.98 (0.46;2.09) | 0.938 |
| ≥60 years [ref] |  |  |  |  |  |  |  |  |  |  |
| Low education | 1.27 (0.59;2.72) | 0.418 | 0.73 (0.29;1.81) | 0.368 | 1.16 (0.68;2.00) | 0.468 | 0.75 (0.40;1.43) | 0.250 | 1.43 (0.88;2.32) | 0.056 |
| Medium education | 1.93 (0.91;4.08) | 0.025 | 1.28 (0.61;2.67) | 0.395 | 0.96 (0.49;1.88) | 0.881 | 0.90 (0.45;1.82) | 0.704 | 1.78 (0.99;3.20) | 0.012 |
| High education [ref] |  |  |  |  |  |  |  |  |  |  |
| High income adequacy | 1.02 (0.56;1.88) | 0.921 | 0.74 (0.33;1.66) | 0.340 | 0.75 (0.42;1.33) | 0.193 | 0.75 (0.41;1.37) | 0.216 | 0.87 (0.55;1.36) | 0.411 |
| Low income adequacy [ref] |  |  |  |  |  |  |  |  |  |  |
| Children at home | 0.47 (0.23;0.93) | 0.004 | 1.24 (0.57;2.72) | 0.476 | 0.72 (0.44;1.19) | 0.092 | 0.41 (0.21;0.78) | <0.001 | 0.63 (0.37;1.06) | 0.023 |
| No children at home [ref] |  |  |  |  |  |  |  |  |  |  |

Abbreviations: UK = United Kingdom; US=United States; AOR= Adjusted odds ratio; CI=Confidence interval.

^a^ Each model was adjusted for year and sociodemographic variables including age, ethnicity, sex, perceived income adequacy, education, and the presence of a child under 18 at home.

^b^ Second listed year is the reference year.

**Table S4**: Perception that sports drinks are “unhealthy”, by country

|  | Australia^a^  (n=1564) | | Canada  (n=1587) | | Mexico  (n=1729) | | UK  (n=1492) | | US  (n=1637) | |
| --- | --- | --- | --- | --- | --- | --- | --- | --- | --- | --- |
|  | AOR(99%CI) | *p* | AOR(99%CI) | *p* | AOR(99%CI) | *p* | AOR(99%CI) | *p* | AOR(99%CI) | *p* |
| 2019 vs 2018^b^ | 1.00 (0.61;1.63) | 0.986 | 0.99 (0.63;1.55) | 0.951 | 1.35 (0.86;2.10) | 0.084 | 1.38 (0.78;2.47) | 0.148 | 1.28 (0.82;2.00) | 0.158 |
| 2020 vs 2018 | 0.85 (0.53;1.34) | 0.350 | 0.81 (0.52;1.27) | 0.233 | 1.43 (0.92;2.21) | 0.037 | 1.09 (0.66;1.81) | 0.666 | 1.00 (0.64;1.57) | 0.992 |
| 2021 vs 2018 | 0.84 (0.53;1.33) | 0.339 | 0.98 (0.62;1.54) | 0.892 | 1.41 (0.91;2.18) | 0.042 | 0.95 (0.58;1.57) | 0.801 | 0.88 (0.56;1.38) | 0.462 |
| 2020 vs 2019 | 0.85 (0.53;1.36) | 0.369 | 0.82 (0.52;1.31) | 0.280 | 1.06 (0.68;1.64) | 0.740 | 0.79 (0.43;1.45) | 0.313 | 0.78 (0.50;1.22) | 0.157 |
| 2021 vs 2019 | 0.85 (0.53;1.35) | 0.361 | 0.99 (0.61;1.59) | 0.943 | 1.05 (0.68;1.62) | 0.786 | 0.69 (0.38;1.26) | 0.110 | 0.69 (0.44;1.08) | 0.032 |
| 2021 vs 2020 | 1.00 (0.64;1.55) | 0.989 | 1.20 (0.75;1.92) | 0.320 | 0.99 (0.64;1.52) | 0.949 | 0.87 (0.51;1.50) | 0.521 | 0.88 (0.56;1.38) | 0.452 |
| Female | 1.82 (1.31;2.53) | <0.001 | 1.55 (1.11;2.15) | 0.001 | 1.58 (1.16;2.15) | <0.001 | 1.51 (1.04;2.21) | 0.005 | 1.82 (1.32;2.52) | <0.001 |
| Male [ref] |  |  |  |  |  |  |  |  |  |  |
| Minority | 0.79 (0.50;1.23) | 0.166 | 0.93 (0.61;1.43) | 0.677 | 0.96 (0.61;1.49) | 0.792 | 0.52 (0.28;0.97) | 0.007 | 0.81 (0.56;1.17) | 0.139 |
| Majority [ref] |  |  |  |  |  |  |  |  |  |  |
| 18-29 years | 0.81 (0.50;1.32) | 0.268 | 1.05 (0.64;1.75) | 0.786 | 1.16 (0.61;2.19) | 0.553 | 0.98 (0.55;1.76) | 0.936 | 1.40 (0.85;2.29) | 0.081 |
| 30-44 years | 1.08 (0.64;1.83) | 0.691 | 1.05 (0.64;1.71) | 0.806 | 1.08 (0.56;2.10) | 0.751 | 1.15 (0.61;2.17) | 0.573 | 1.33 (0.81;2.18) | 0.144 |
| 45-59 years | 1.61 (1.02;2.55) | 0.008 | 1.02 (0.66;1.59) | 0.905 | 0.89 (0.45;1.75) | 0.659 | 1.14 (0.65;2.01) | 0.538 | 1.34 (0.86;2.09) | 0.089 |
| ≥60 years [ref] |  |  |  |  |  |  |  |  |  |  |
| Low education | 0.80 (0.52;1.24) | 0.195 | 0.40 (0.26;0.60) | <0.001 | 0.72 (0.49;1.08) | 0.036 | 0.53 (0.34;0.82) | <0.001 | 0.63 (0.45;0.88) | 0.001 |
| Medium education | 0.92 (0.60;1.39) | 0.589 | 0.75 (0.53;1.07) | 0.038 | 0.72 (0.44;1.19) | 0.096 | 0.69 (0.44;1.08) | 0.031 | 0.79 (0.55;1.13) | 0.088 |
| High education [ref] |  |  |  |  |  |  |  |  |  |  |
| High income adequacy | 1.18 (0.84;1.67) | 0.213 | 1.06 (0.76;1.49) | 0.637 | 0.98 (0.65;1.48) | 0.916 | 1.01 (0.68;1.51) | 0.950 | 1.06 (0.77;1.46) | 0.660 |
| Low income adequacy [ref] |  |  |  |  |  |  |  |  |  |  |
| Children at home | 0.82 (0.54;1.25) | 0.224 | 0.76 (0.50;1.17) | 0.105 | 0.64 (0.46;0.89) | 0.001 | 0.60 (0.37;0.98) | 0.007 | 0.63 (0.41;0.96) | 0.005 |
| No children at home [ref] |  |  |  |  |  |  |  |  |  |  |

Abbreviations: UK = United Kingdom; US=United States; AOR= Adjusted odds ratio; CI=Confidence interval.

^a^ Each model was adjusted for year and sociodemographic variables including age, ethnicity, sex, perceived income adequacy, education, and the presence of a child under 18 at home.

^b^ Second listed year is the reference year.

**Table S5**: Perception that chocolate milk is “unhealthy”, by country

|  | Australia^a^  (n=1628) | | Canada  (n=1610) | | Mexico  (n=1577) | | UK  (n=1589) | | US  (n=1644) | |
| --- | --- | --- | --- | --- | --- | --- | --- | --- | --- | --- |
|  | AOR(99%CI) | *p* | AOR(99%CI) | *p* | AOR(99%CI) | *p* | AOR(99%CI) | *p* | AOR(99%CI) | *p* |
| 2019 vs 2018^b^ | 0.96 (0.58;1.57) | 0.819 | 0.89 (0.58;1.38) | 0.497 | 1.17 (0.74;1.85) | 0.377 | 0.97 (0.60;1.55) | 0.857 | 1.65 (1.04;2.60) | 0.005 |
| 2020 vs 2018 | 0.82 (0.51;1.32) | 0.274 | 0.97 (0.63;1.49) | 0.858 | 1.94 (1.24;3.05) | <0.001 | 1.26 (0.81;1.96) | 0.179 | 1.25 (0.81;1.94) | 0.189 |
| 2021 vs 2018 | 0.63 (0.38;1.02) | 0.013 | 1.09 (0.70;1.68) | 0.626 | 2.69 (1.70;4.26) | <0.001 | 0.79 (0.52;1.21) | 0.159 | 1.41 (0.89;2.22) | 0.053 |
| 2020 vs 2019 | 0.85 (0.53;1.37) | 0.385 | 1.09 (0.71;1.66) | 0.607 | 1.66 (1.06;2.61) | 0.004 | 1.30 (0.80;2.13) | 0.165 | 0.76 (0.49;1.19) | 0.114 |
| 2021 vs 2019 | 0.65 (0.40;1.06) | 0.024 | 1.22 (0.79;1.87) | 0.241 | 2.30 (1.45;3.64) | <0.001 | 0.82 (0.51;1.32) | 0.275 | 0.86 (0.54;1.36) | 0.385 |
| 2021 vs 2020 | 0.77 (0.48;1.22) | 0.140 | 1.12 (0.73;1.71) | 0.497 | 1.38 (0.88;2.17) | 0.065 | 0.63 (0.40;0.98) | 0.008 | 1.13 (0.72;1.76) | 0.497 |
| Female | 1.91 (1.35;2.70) | <0.001 | 1.70 (1.26;2.31) | <0.001 | 1.43 (1.04;1.97) | 0.004 | 1.30 (0.94;1.79) | 0.036 | 1.39 (1.00;1.92) | 0.010 |
| Male [ref] |  |  |  |  |  |  |  |  |  |  |
| Minority | 0.66 (0.42;1.02) | 0.014 | 0.86 (0.58;1.27) | 0.315 | 0.72 (0.46;1.11) | 0.051 | 0.47 (0.28;0.80) | <0.001 | 0.94 (0.65;1.36) | 0.671 |
| Majority [ref] |  |  |  |  |  |  |  |  |  |  |
| 18-29 years | 1.24 (0.74;2.08) | 0.285 | 1.61 (0.99;2.62) | 0.012 | 2.65 (1.22;5.76) | 0.001 | 1.43 (0.87;2.37) | 0.065 | 1.57 (0.96;2.55) | 0.017 |
| 30-44 years | 1.35 (0.80;2.27) | 0.142 | 1.11 (0.72;1.70) | 0.531 | 1.83 (0.83;4.05) | 0.049 | 1.12 (0.67;1.86) | 0.567 | 1.80 (1.09;2.96) | 0.002 |
| 45-59 years | 1.65 (1.03;2.64) | 0.006 | 0.85 (0.56;1.30) | 0.336 | 1.42 (0.64;3.14) | 0.256 | 1.17 (0.74;1.84) | 0.384 | 1.30 (0.85;2.00) | 0.108 |
| ≥60 years [ref] |  |  |  |  |  |  |  |  |  |  |
| Low education | 0.89 (0.57;1.39) | 0.500 | 0.60 (0.41;0.88) | 0.001 | 0.74 (0.49;1.12) | 0.064 | 0.43 (0.30;0.62) | <0.001 | 0.74 (0.52;1.06) | 0.031 |
| Medium education | 1.10 (0.72;1.69) | 0.566 | 0.78 (0.56;1.09) | 0.057 | 0.89 (0.52;1.53) | 0.580 | 0.70 (0.48;1.03) | 0.019 | 0.99 (0.67;1.46) | 0.961 |
| High education [ref] |  |  |  |  |  |  |  |  |  |  |
| High income adequacy | 0.98 (0.69;1.39) | 0.885 | 1.06 (0.77;1.45) | 0.649 | 0.86 (0.57;1.30) | 0.347 | 0.91 (0.65;1.27) | 0.475 | 0.86 (0.61;1.22) | 0.264 |
| Low income adequacy [ref] |  |  |  |  |  |  |  |  |  |  |
| Children at home | 0.82 (0.52;1.27) | 0.238 | 0.75 (0.51;1.10) | 0.052 | 0.88 (0.62;1.24) | 0.338 | 0.72 (0.47;1.08) | 0.037 | 0.62 (0.41;0.94) | 0.003 |
| No children at home [ref] |  |  |  |  |  |  |  |  |  |  |

Abbreviations: UK = United Kingdom; US=United States; AOR= Adjusted odds ratio; CI=Confidence interval.

^a^ Each model was adjusted for year and sociodemographic variables including age, ethnicity, sex, perceived income adequacy, education, and the presence of a child under 18 at home.

^b^ Second listed year is the reference year.

**Table S6**: Perception that iced tea is “unhealthy” by country

|  | Australia^a^  (n=1627) | | Canada  (n=1671) | | Mexico  (n=1659) | | UK  (n=1537) | | US  (n=1600) | |
| --- | --- | --- | --- | --- | --- | --- | --- | --- | --- | --- |
|  | AOR(99%CI) | *p* | AOR(99%CI) | *p* | AOR(99%CI) | *p* | AOR(99%CI) | *p* | AOR(99%CI) | *p* |
| 2019 vs 2018^b^ | 0.97 (0.61;1.55) | 0.881 | 1.01 (0.62;1.66) | 0.958 | 1.24 (0.82;1.87) | 0.186 | 1.16 (0.75;1.80) | 0.382 | 0.88 (0.56;1.37) | 0.457 |
| 2020 vs 2018 | 0.80 (0.52;1.22) | 0.171 | 1.10 (0.67;1.79) | 0.627 | 2.23 (1.41;3.54) | <0.001 | 0.99 (0.65;1.51) | 0.941 | 0.93 (0.60;1.44) | 0.668 |
| 2021 vs 2018 | 0.83 (0.53;1.28) | 0.256 | 1.09 (0.66;1.80) | 0.662 | 1.79 (1.15;2.76) | 0.001 | 0.97 (0.62;1.51) | 0.843 | 0.99 (0.64;1.54) | 0.958 |
| 2020 vs 2019 | 0.82 (0.53;1.27) | 0.239 | 1.09 (0.67;1.76) | 0.664 | 1.81 (1.13;2.87) | 0.001 | 0.85 (0.54;1.34) | 0.359 | 1.06 (0.67;1.67) | 0.756 |
| 2021 vs 2019 | 0.85 (0.54;1.33) | 0.344 | 1.08 (0.65;1.78) | 0.699 | 1.44 (0.93;2.25) | 0.033 | 0.83 (0.52;1.34) | 0.319 | 1.13 (0.71;1.78) | 0.503 |
| 2021 vs 2020 | 1.04 (0.69;1.56) | 0.825 | 0.99 (0.61;1.63) | 0.973 | 0.80 (0.50;1.29) | 0.229 | 0.98 (0.62;1.54) | 0.901 | 1.07 (0.68;1.68) | 0.716 |
| Female | 1.55 (1.14;2.12) | <0.001 | 2.09 (1.46;2.98) | <0.001 | 1.82 (1.33;2.49) | <0.001 | 1.44 (1.05;1.98) | 0.003 | 1.31 (0.94;1.81) | 0.035 |
| Male [ref] |  |  |  |  |  |  |  |  |  |  |
| Minority | 0.97 (0.63;1.51) | 0.870 | 1.10 (0.70;1.73) | 0.606 | 1.02 (0.66;1.57) | 0.903 | 0.99 (0.57;1.72) | 0.954 | 0.99 (0.69;1.42) | 0.945 |
| Majority [ref] |  |  |  |  |  |  |  |  |  |  |
| 18-29 years | 1.38 (0.85;2.23) | 0.085 | 1.14 (0.67;1.96) | 0.518 | 1.57 (0.80;3.06) | 0.083 | 1.37 (0.86;2.20) | 0.082 | 1.13 (0.70;1.83) | 0.519 |
| 30-44 years | 1.47 (0.93;2.34) | 0.031 | 1.53 (0.90;2.60) | 0.041 | 1.50 (0.76;2.99) | 0.125 | 1.79 (1.10;2.91) | 0.002 | 1.08 (0.66;1.77) | 0.689 |
| 45-59 years | 1.50 (0.99;2.28) | 0.013 | 1.48 (0.91;2.39) | 0.036 | 0.87 (0.44;1.76) | 0.619 | 1.20 (0.77;1.86) | 0.294 | 1.26 (0.81;1.96) | 0.177 |
| ≥60 years [ref] |  |  |  |  |  |  |  |  |  |  |
| Low education | 0.76 (0.50;1.14) | 0.079 | 0.47 (0.31;0.72) | <0.001 | 0.64 (0.43;0.96) | 0.005 | 0.52 (0.37;0.75) | <0.001 | 0.57 (0.40;0.82) | <0.001 |
| Medium education | 0.96 (0.64;1.44) | 0.799 | 0.83 (0.57;1.22) | 0.218 | 0.80 (0.49;1.30) | 0.228 | 0.70 (0.49;1.01) | 0.011 | 0.87 (0.60;1.26) | 0.321 |
| High education [ref] |  |  |  |  |  |  |  |  |  |  |
| High income adequacy | 0.99 (0.71;1.38) | 0.942 | 1.08 (0.75;1.56) | 0.565 | 1.01 (0.67;1.51) | 0.968 | 0.98 (0.71;1.36) | 0.894 | 1.05 (0.74;1.49) | 0.703 |
| Low income adequacy [ref] |  |  |  |  |  |  |  |  |  |  |
| Children at home | 0.91 (0.62;1.35) | 0.553 | 1.00 (0.64;1.56) | 0.987 | 0.78 (0.55;1.11) | 0.066 | 0.84 (0.56;1.27) | 0.284 | 0.80 (0.53;1.19) | 0.150 |
| No children at home [ref] |  |  |  |  |  |  |  |  |  |  |

Abbreviations: UK = United Kingdom; US=United States; AOR= Adjusted odds ratio; CI=Confidence interval.

^a^ Each model was adjusted for year and sociodemographic variables including age, ethnicity, sex, perceived income adequacy, education, and the presence of a child under 18 at home.

^b^ Second listed year is the reference year.

**Table S7**: Perception that specialty coffee is “unhealthy”, by country

|  | Australia^a^  (n=1668) | | Canada  (n=1657) | | Mexico  (n=1631) | | UK  (n=1592) | | US  (n=1645) | |
| --- | --- | --- | --- | --- | --- | --- | --- | --- | --- | --- |
|  | AOR(99%CI) | *p* | AOR(99%CI) | *p* | AOR(99%CI) | *p* | AOR(99%CI) | *p* | AOR(99%CI) | *p* |
| 2019 vs 2018^b^ | 1.04 (0.64;1.70) | 0.823 | 1.37 (0.81;2.32) | 0.126 | 0.76 (0.49;1.18) | 0.109 | 1.24 (0.75;2.07) | 0.274 | 1.55 (0.92;2.62) | 0.032 |
| 2020 vs 2018 | 1.12 (0.68;1.85) | 0.559 | 1.49 (0.84;2.64) | 0.076 | 1.68 (1.07;2.65) | 0.003 | 0.92 (0.59;1.45) | 0.654 | 1.33 (0.81;2.19) | 0.139 |
| 2021 vs 2018 | 0.86 (0.53;1.39) | 0.420 | 1.09 (0.63;1.90) | 0.683 | 1.36 (0.86;2.14) | 0.084 | 0.90 (0.57;1.42) | 0.556 | 1.29 (0.78;2.12) | 0.192 |
| 2020 vs 2019 | 1.07 (0.65;1.77) | 0.712 | 1.09 (0.62;1.91) | 0.706 | 2.21 (1.41;3.46) | <0.001 | 0.74 (0.44;1.27) | 0.152 | 0.86 (0.51;1.45) | 0.451 |
| 2021 vs 2019 | 0.82 (0.51;1.33) | 0.300 | 0.80 (0.46;1.38) | 0.289 | 1.78 (1.13;2.80) | 0.001 | 0.73 (0.43;1.24) | 0.121 | 0.83 (0.49;1.41) | 0.369 |
| 2021 vs 2020 | 0.77 (0.47;1.25) | 0.163 | 0.73 (0.41;1.33) | 0.180 | 0.81 (0.50;1.29) | 0.239 | 0.98 (0.61;1.57) | 0.895 | 0.97 (0.59;1.60) | 0.868 |
| Female | 1.73 (1.22;2.44) | <0.001 | 1.92 (1.28;2.88) | <0.001 | 1.39 (1.01;1.91) | 0.008 | 1.39 (0.98;1.96) | 0.015 | 1.84 (1.28;2.67) | <0.001 |
| Male [ref] |  |  |  |  |  |  |  |  |  |  |
| Minority | 0.54 (0.34;0.86) | 0.001 | 0.72 (0.45;1.15) | 0.070 | 0.78 (0.49;1.25) | 0.174 | 0.45 (0.26;0.79) | <0.001 | 0.58 (0.38;0.88) | 0.001 |
| Majority [ref] |  |  |  |  |  |  |  |  |  |  |
| 18-29 years | 2.18 (1.24;3.81) | <0.001 | 2.05 (1.08;3.87) | 0.004 | 1.64 (0.81;3.28) | 0.069 | 1.28 (0.74;2.21) | 0.251 | 0.73 (0.42;1.27) | 0.143 |
| 30-44 years | 1.73 (1.05;2.87) | 0.005 | 1.78 (0.99;3.22) | 0.012 | 1.48 (0.73;3.00) | 0.148 | 1.16 (0.69;1.95) | 0.473 | 0.82 (0.46;1.45) | 0.372 |
| 45-59 years | 1.37 (0.87;2.16) | 0.077 | 1.86 (1.06;3.27) | 0.005 | 1.19 (0.58;2.46) | 0.536 | 1.65 (0.98;2.80) | 0.014 | 1.14 (0.67;1.93) | 0.538 |
| ≥60 years [ref] |  |  |  |  |  |  |  |  |  |  |
| Low education | 1.06 (0.68;1.64) | 0.754 | 1.51 (0.94;2.43) | 0.025 | 0.86 (0.57;1.30) | 0.342 | 0.65 (0.44;0.96) | 0.004 | 0.73 (0.50;1.07) | 0.033 |
| Medium education | 1.09 (0.69;1.71) | 0.643 | 2.02 (1.27;3.21) | <0.001 | 1.22 (0.75;1.99) | 0.287 | 1.15 (0.77;1.71) | 0.375 | 0.71 (0.46;1.10) | 0.044 |
| High education [ref] |  |  |  |  |  |  |  |  |  |  |
| High income adequacy | 1.09 (0.75;1.58) | 0.543 | 1.20 (0.79;1.82) | 0.265 | 0.93 (0.62;1.41) | 0.665 | 0.95 (0.66;1.37) | 0.718 | 0.72 (0.48;1.06) | 0.029 |
| Low income adequacy [ref] |  |  |  |  |  |  |  |  |  |  |
| Children at home | 0.91 (0.59;1.40) | 0.569 | 0.71 (0.42;1.18) | 0.081 | 0.96 (0.68;1.34) | 0.724 | 0.72 (0.47;1.10) | 0.047 | 0.48 (0.31;0.75) | <0.001 |
| No children at home [ref] |  |  |  |  |  |  |  |  |  |  |

Abbreviations: UK = United Kingdom; US=United States; AOR= Adjusted odds ratio; CI=Confidence interval.

^a^ Each model was adjusted for year and sociodemographic variables including age, ethnicity, sex, perceived income adequacy, education, and the presence of a child under 18 at home.

^b^ Second listed year is the reference year.
